# Structural basis for the complete resistance of the human prion protein mutant G127V to prion disease

**DOI:** 10.1038/s41598-018-31394-6

**Published:** 2018-09-04

**Authors:** Zhen Zheng, Meilan Zhang, Yongheng Wang, Rongsheng Ma, Chenyun Guo, Liubin Feng, Jihui Wu, Hongwei Yao, Donghai Lin

**Affiliations:** 1 MOE Key Laboratory of Spectrochemical Analysis & Instrumentation, Key Laboratory of Chemical Biology of Fujian Province, College of Chemistry and Chemical Engineering, Xiamen University, Xiamen, 361005 China; 2 School of Pharmaceutical Sciences, Sun Yat-sen University, Guangzhou, 510006 China; 3 School of Life Sciences, University of Science and Technology of China, Hefei, 230026 China

## Abstract

Prion diseases are caused by the propagation of misfolded cellular prion proteins (PrPs). A completely prion disease-resistant genotype, V127M129, has been identified in Papua New Guinea and verified in transgenic mice. To disclose the structural basis of the disease-resistant effect of the G127V mutant, we determined and compared the structural and dynamic features of the G127V-mutated human PrP (residues 91–231) and the wild-type PrP in solution. HuPrP(G127V) contains α1, α2 and α3 helices and a stretch-strand (SS) pattern comprising residues Tyr128-Gly131 (SS1) and Val161-Arg164 (SS2), with extending atomic distances between the SS1 and SS2 strands, and a structural rearrangement of the Tyr128 side chain due to steric hindrance of the larger hydrophobic side chain of Val127. The extended α1 helix gets closer to the α2 and α3 helices. NMR dynamics analysis revealed that Tyr128, Gly131 and Tyr163 underwent significant conformational exchanges. Molecular dynamics simulations suggest that HuPrP(G127V) prevents the formation of stable β-sheets and dimers. Unique structural and dynamic features potentially inhibit the conformational conversion of the G127V mutant. This work is beneficial for understanding the molecular mechanisms underlying the complete resistance of the G127V mutant to prion disease and for developing new therapeutics for prion disease.

## Introduction

Prion diseases, the notorious transmissible spongiform encephalopathies (TSEs), are infectious and fatal central nervous system (CNS) degenerative diseases in some mammals^1^. In humans, prion diseases manifest as a variety of clinical symptoms: Creutzfeldt-Jakob diseases (CJD, including *s*poradic, *i*atrogenic, *v*ariant, and *f*amilial/*g*enetic CJD)^2–5^, Gerstmann-Sträussler-Scheinker syndrome (GSS)^2,6^, fatal familial insomnia (FFI)^2,5,7^ and Kuru^2,8,9^. These diseases are caused by the propagation of the insoluble scrapie isoform of the prion protein (PrP^Sc^), a β-sheet-rich form, which is originated from the normal cellular prion protein (PrP^C^) through conformational conversion and is resistant to proteinases^2,10,11^. The α-helical PrP^C^ is encoded by the endogenous prion protein gene (*PRNP*)^1^ and acts as an agonistic ligand of the Adgrg6^12^. To date, more than 50 pathogenic point mutations of *PRNP* have been associated with a variety of prion diseases^13–15^. Fortunately, two frail *s*CJD-resistant mutants, V209M^13,16^ and E219K^17–19^, have been clinically observed, and their disease-resistance mechanisms have been addressed. Furthermore, a completely prion disease-resistant mutant G127V has also been identified in the bodies of Papua New Guinea’s Fore tribe and verified in transgenic mice studies^20,21^. However, the underlying disease-resistance mechanisms remain elusive.

Protein structure and dynamics primarily decide function. Structural biology is used extensively to exploit the molecular mechanisms of protein function. Previous work has shown that wild-type (WT) human prion protein (HuPrP) contains an N-terminal random coil (residues 23–124) and a C-terminal globular domain (residues 125–231)^22^ associated with many pathogenic and protective mutations^13–15^. The three-dimensional (3D) structure of the C-terminal domain in the WT protein is comprised of three α-helices (α1: 144–154, α2: 173–194, α3: 200–228), two short β-strands (β1: 128–131, β2: 161–164) and a disulfide bridge (Cys179-Cys214) even though under different pH conditions^16,22–24^. Almost all the HuPrP mutants adopt similar 3D structures to the WT protein^16,22–29^.

Previous ^15^N backbone dynamic studies of WT HuPrP show that the rigid globular C-terminal core has lower ^15^N longitudinal relaxation rates, higher ^15^N transverse relaxation rates, and much more positive {^1^H}-^15^N heteronuclear steady-state nuclear Overhauser effects (NOEs) than the N-terminal random coil^23,29–32^. Conformational exchanges on the μs-ms timescale were exhibited by the residues at or near the short anti-parallel β-sheet^30^ but were too small to be detected by Carr-Purcell-Meiboom-Gill (CPMG) relaxation dispersion (RD) experiments^29–31^. Due to the dramatic flexibility of the long N-terminal coil of the prion protein, the conventional model-free approach cannot be used to analyse NMR dynamics data^29,30,32^.

Based on the crystal structure of the WT HuPrP, 3D domain swapping with an intermolecular β-sheet may be an important step in the conversion of PrP^C^ to PrP^Sc^^33,34^. However, growing evidence has shown that the β1- and β2-strands, especially the β1-strand, and the interchain between them might trigger PrP^Sc^ propagation^34–36^. A series of β1-strands can form steric zippers with different packing geometries depending on the presence of either the Met129 or Val129 *PRNP* alleles^35,36^, even though the monomeric structures of the two genotypes do not show striking differences^34,37,38^. Importantly, molecular dynamics (MD) simulations confirmed that the slight protective heterozygosity of 129MV assembled extremely unstable intermolecular β-sheets due to the Val129 side chain, conflicting with spatially adjacent residues^39–42^. The different genotypes of residue 129 could cause the different stabilities of the β-sheet^43–45^, the different conformational conversion of the N-terminal flexible segment^46^, and the intrinsic conformational heterogeneity of the α1 helix^47^. For instance, D178N/M129 associated with FFI^5,7^ and D178N/V129 related to *f*CJD^5,7^ showed different intermolecular tetramers in crystal structures^34^, different dynamic features^43,44,48^ and different rates of the conversion to amyloid fibrils which were larger than WT *in vitro*^39^.

Furthermore, the frailly protective mutant HuPrP(V209M) against *s*CJD changes the geometric packing of the α1 and α3 helices and impairs the tendency of amyloid fibre formation^13,16^. The V209M mutation decreased the fibrillization rate relative to WT *in vitro*^16^. Quite differently, although the V210I mutant alters the geometry of the α2 and α3 helices^27,49^, it is fully susceptible to *g*CJD^50^. In addition, another frailly protective mutant against *s*CJD, HuPrP(E219K)^17–19^, has a slightly altered 3D structure, changed backbone dynamics, and redistributed surface electrostatic potentials^23^. The mutated Lys219 residue exhibits one of the highest ^15^N R_2_ rates, indicative of decreased backbone flexibility or/and increased conformational exchange on the μs-ms timescale^23^. The incompatible structures and dynamics of the heterozygous 219EK mutant potentially prevent hetero-dimerization^51^, which resists the development of *s*CJD^17–19,23,51^. Interestingly, even though the E200K mutant also redistributed the surface electrostatic potentials, it still leads to *g*CJD^19,26,27,52^. Thus, whether or not the alterations in the geometric packing of the α helices or the surface electrostatic potentials resist disease requires further studies.

In the completely disease-resistant G127V mutant, Val127 was observed exclusively on a Met129 *PRNP* allele^21^. In transgenic mice experiments, heterozygous 127 GV mice were able to resist Kuru and all of the CJDs except *v*CJD. Moreover, the protein expression ratios of the Gly127 to Val127 genotypes greatly affected the ability to resist prion disease. More significantly, homozygous 127VV mice were entirely resistant to all the prion diseases^21^. To identify the molecular mechanisms underlying the significant disease-resistant effect of the G127V mutant, we determined solution structures of both the recombinant HuPrP (residues 91–231) with the V127M129 genotype (termed HuPrP(G127V)) and the WT HuPrP with a G127M129 genotype (termed WT HuPrP) at pH 4.5^22^. We performed NMR dynamics analysis and implemented MD simulations on the determined structures for the two proteins. We observed several significant differences in the structural and dynamic properties of the two proteins. These results provide novel insight into the molecular mechanisms of the disease-resistant effect of the G127V mutant. This work may be of benefit to both a mechanistic understanding of prion propagation and the development of effective therapeutics.

## Results

### Resonance assignments of HuPrP(G127V) and WT HuPrP

**Figure 1:**
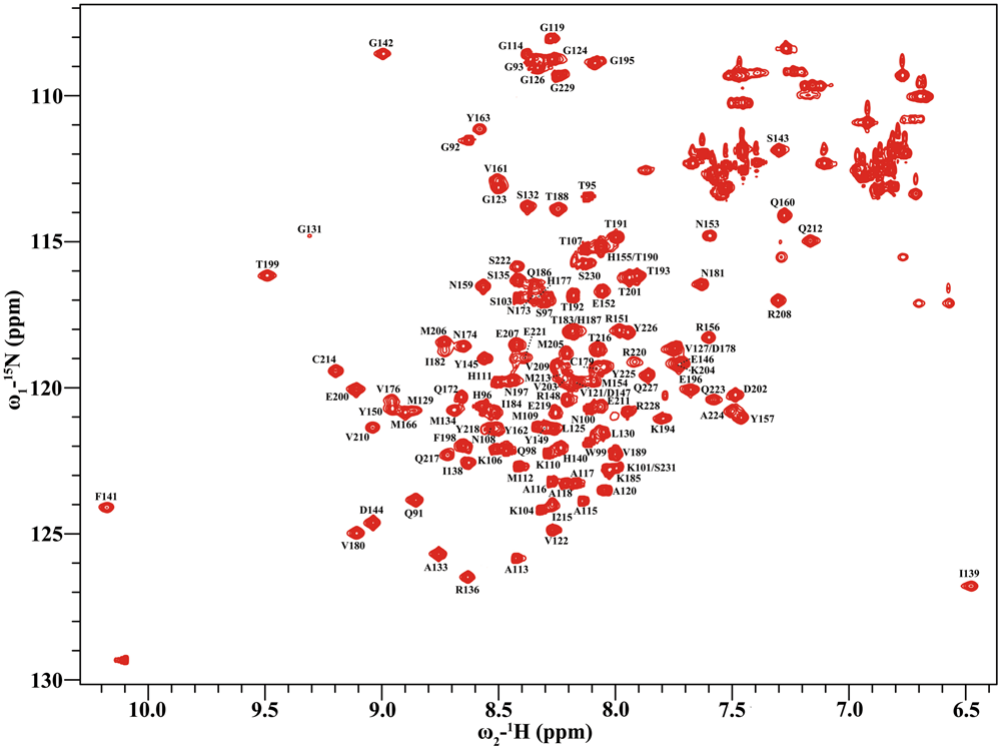
2D ^1^H-^15^N HSQC spectrum of ^15^N-labelled HuPrP(G127V). Assignments of the backbone N-H resonances are identified with the one letter amino acid codes and the sequence number. The spectrum was recorded at a magnetic field strength of 19.97 T.

The well-dispersed ^1^H-^15^N HSQC spectra illustrate that both HuPrP(G127V) and WT HuPrP adopt well-folded structures (Figs 1 and S1). All resonance assignments were verified by 3D ^15^N-edited NOSEY-HSQC and ^13^C-edited NOESY-HSQC spectra. Overall, 91% resonances were assigned for the two proteins. In total, for the 136 backbone N-H resonances of HuPrP (residues 91–231) (141 residues minus 5 prolines), 127 and 128 resonances were unambiguously assigned to HuPrP(G127V) (BMRB ID: 27259) and WT HuPrP (BMRB ID: 27264), respectively. Backbone N-H resonances could not be obtained for Gly94, Arg164, Asp167, Glu168, Tyr169, Ser170, Asn171 and Phe175. Notably, the amine resonance of Tyr128 disappeared from the HSQC spectrum of HuPrP(G127V), but was visible as an isolated peak in that of WT HuPrP (Figs 1 and S1). The ^1^H-^15^N HSQC spectra between the mutant and WT proteins illustrate that the G127V mutant introduced notably changed chemical shifts for Met129, Val161, and Tyr162, with distinct peak broadening for Gly131 (Figs 1 and S1).

### Solution Structures of HuPrP(G127V) and WT HuPrP at pH 4.5

Based on the resonance assignments and experimentally conformational restraints, we determined the solution structures of HuPrP(G127V) (PDB ID: 5YJ4) and WT HuPrP (PDB ID: 5YJ5) at pH 4.5 and 298K. The complete structural statistics are summarized in Table S1.

**Figure 2:**
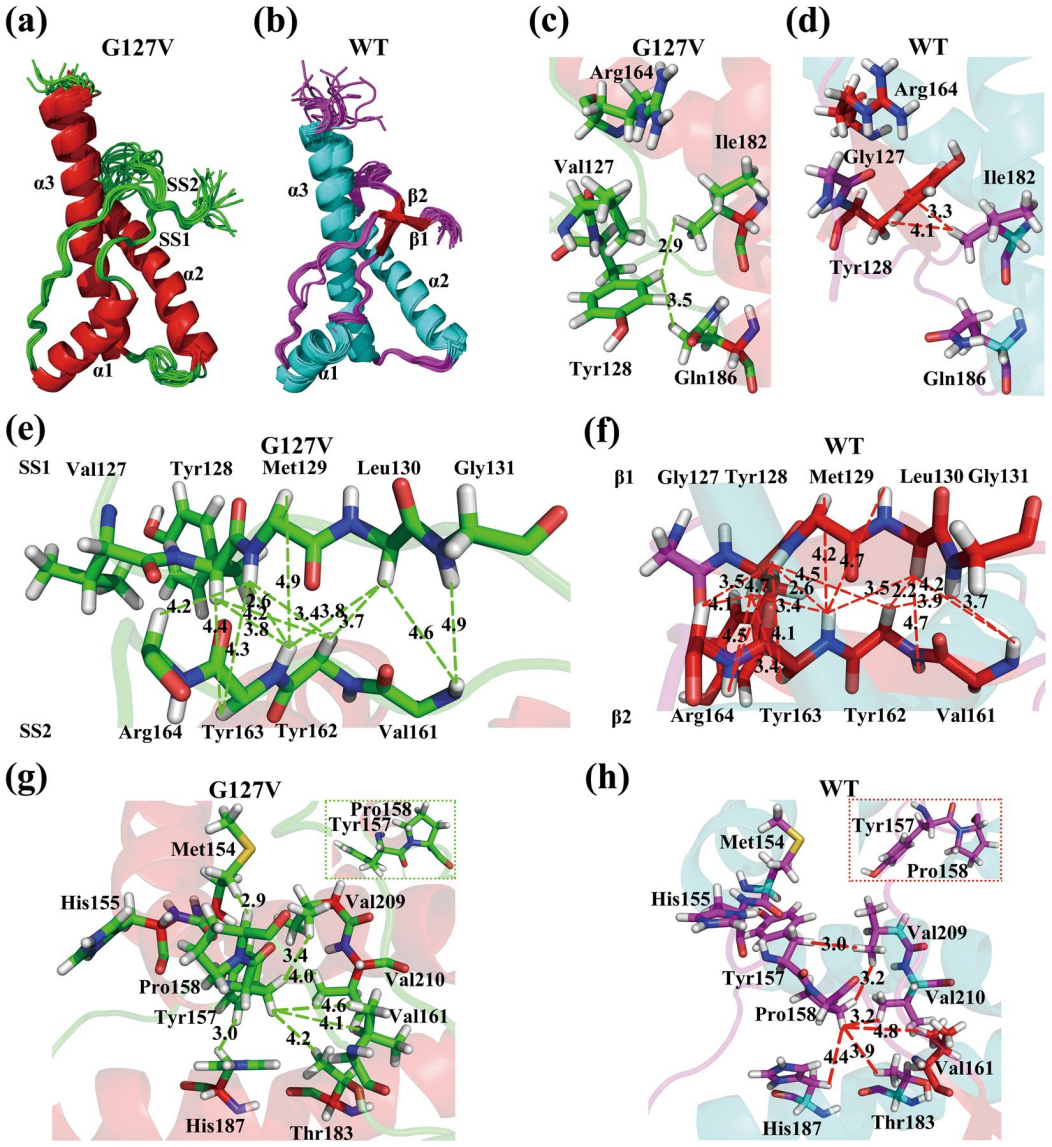
Structural analysis of HuPrP(G127V) and WT HuPrP. **(a**,**b)** Cartoons of the 20 lowest-energy conformers for the mutant and WT proteins. **(c**,**d)** Details of the structural alteration at Tyr128 resulting from the G127V mutation (showing only d_Hε/Tyr128-Hγ/Ile182_ and d_Hε/Tyr128-Hγ/Gln186_ < 5.0 Å). **(e**,**f)** Distances (d_αα_, d_αN_, d_NN_ < 5.0 Å) between the two strands (stretch-strands for G127V, β-strands for WT). **(g**,**h)** Local conformational alterations caused by the change of the dihedral angle psi(N-Cα-C-N) in Tyr157. These results demonstrate that the C-terminal structural cores of the mutant and WT proteins adopt different conformations.

Similar to WT HuPrP, the solution structure of HuPrP(G127V) (Fig. 2a,b) consisted of the N-terminal flexible segment (Gln91-Gly124) and the C-terminal structural core (Leu125-Ser231) containing three α helices (α1: Asp144-Arg156, α2: Gln172-Lys194, α3: Glu200-Arg228) and a disulfide bond (Cys179-Cys214). The backbone root-mean-square deviation (RMSD) between the average structures of the mutant and WT proteins was 2.27 Å (Fig. S2). The G127V mutant formed a *s*tretch-*s*trand (SS) pattern with two segments (SS1: Tyr128-Gly131; SS2: Val161-Arg164), while the WT protein formed a stable β-sheet with two strands (β1: Tyr128-Gly131; β2: Val161-Arg164). Compared with WT HuPrP, HuPrP(G127V) exhibited unique structural characteristics (Fig. S2a,d), including the following: (I) a smaller distance between the α1 helix and α2/α3 helices; (II) a larger curvature of the α1-SS2 loop (Tyr157-Gln160); (III) a more flexible SS2-α2 loop (Pro165-Asn171) (backbone RMSD of 1.28 Å in the mutant vs. 0.88 Å in WT HuPrP); and (IV) a bigger bend in the α2 helix such that its C-terminal is closer to the N-terminal end of the α3 helix.

In HuPrP(G127V), the hydrophilic side chain of Tyr128 is rotated sharply so that its dihedral angle, chi (N-Cα-Cβ-Cγ), is reduced from 90° to 0° in WT HuPrP. This structural rearrangement might be introduced by the steric hindrance of the relatively larger hydrophobic side chain of Val127. This rotation pushes the phenyl ring of Tyr128 away from Ile182 and brings it closer to Gln186 (Fig. 2c,d). Although the HN_Met129_-HN_Tyr163_ distance is identical for both structures (2.6 ± 0.1 Å), the Hα_Leu130_-Hα_Tyr162_ distance is larger in the mutant (3.7 ± 0.2 Å vs. 2.2 ± 0.2 Å), as shown in Fig. 2e,f. The backbone atomic distances between SS1 and SS2 or between β1 and β2 are summarized in Table S2.

In the mutant protein, the C-terminal end of the α1 helix is extended to Arg156 (Figs 2a,b and S2a,b). In this configuration, the Arg156 side chain is closer to Thr190 and Thr191 at the C-terminal end of the α2 helix (Fig. S2c,d). In the α1-SS2 loop of HuPrP(G127V), the dihedral angle psi(N-Cα-C-N) of Tyr157 is nearly 60°, causing the pyrrolidine of Pro158 to retroflex approximately 180° (Fig. 2g,h). This alteration changes the atomic distances between Tyr157, Pro158, Val209 and Val210. Tyr157 becomes close to two α3-located residues, Val209 and Val210, and Pro158 moves away from Val209 and Val210. The retroflexion of the Pro158 side chain increases the curvature of the α1-SS2 loop in the mutant protein compared with the α1-β2 loop (His155-Gln160) in the WT (Fig. 2g,h). Additionally, atomic distances between SS2 and the disulfide bridge in the mutant are shorter than those between β2 and the disulfide bridge in the WT (Fig. S2g,h).

Furthermore, the G127V mutant also leads to a redistribution of the surface electrostatic potentials of the protein (Fig. S3). HuPrP(G127V) exhibits neutral potentials near residues Gly126-Ser135, while WT HuPrP shows positive potentials in this segment, except for Met129-Gly131. Additionally, compared with the WT protein, the mutant displays more positive potentials in the region near Arg146 and Arg151 of the α1 helix, and more negative potentials on the N-termini of the α1 and α3 helices.

The two different structures were calculated from their own different NOESY restraints originated from 3D ^15^N-edited NOESY-HSQC and ^13^C-edited NOESY-HSQC spectra (Fig. S4, Table S3). Several ^1^H-^1^H NOE peaks were missing, and many peaks were weaker in the SS segments from the HuPrP(G127V) than in those from the WT HuPrP (Fig. S4, Table S3). Furthermore, the structural differences were validated by backbone amide residual dipolar couplings (RDCs) measured from 2D ^1^H-^15^N IPAP-HSQC spectra (Figs S5 and S6). As indicated by the Q-values, the experimental RDCs from HuPrP(G127V) fitted better with the HuPrP(G127V) structure (Q = 0.532) than those with the WT HuPrP structure (Q = 0.798) and vice versa for the RDCs of the WT HuPrP (G127V vs WT: 0.856 vs 0.564)(Figs S5 and S6). In addition, the differences were confirmed with H/D exchanges based on 2D ^1^H-^15^N Fast-HSQC experiments (Figs S7 and S8). Remarkably, the amide proton of Gly131 in the SS segments from the HuPrP(G127V) was exchanged completely with D_2_O and became invisible in the HSQC spectrum than in those from the WT HuPrP (Figs S7 and S8). However, the amide protons of Met154 and His155 in the extended α1 helix from the HuPrP(G127V) became more stable than in those from the WT HuPrP (Figs S7 and S8).

### Backbone amide relaxation analysis

**Figure 3:**
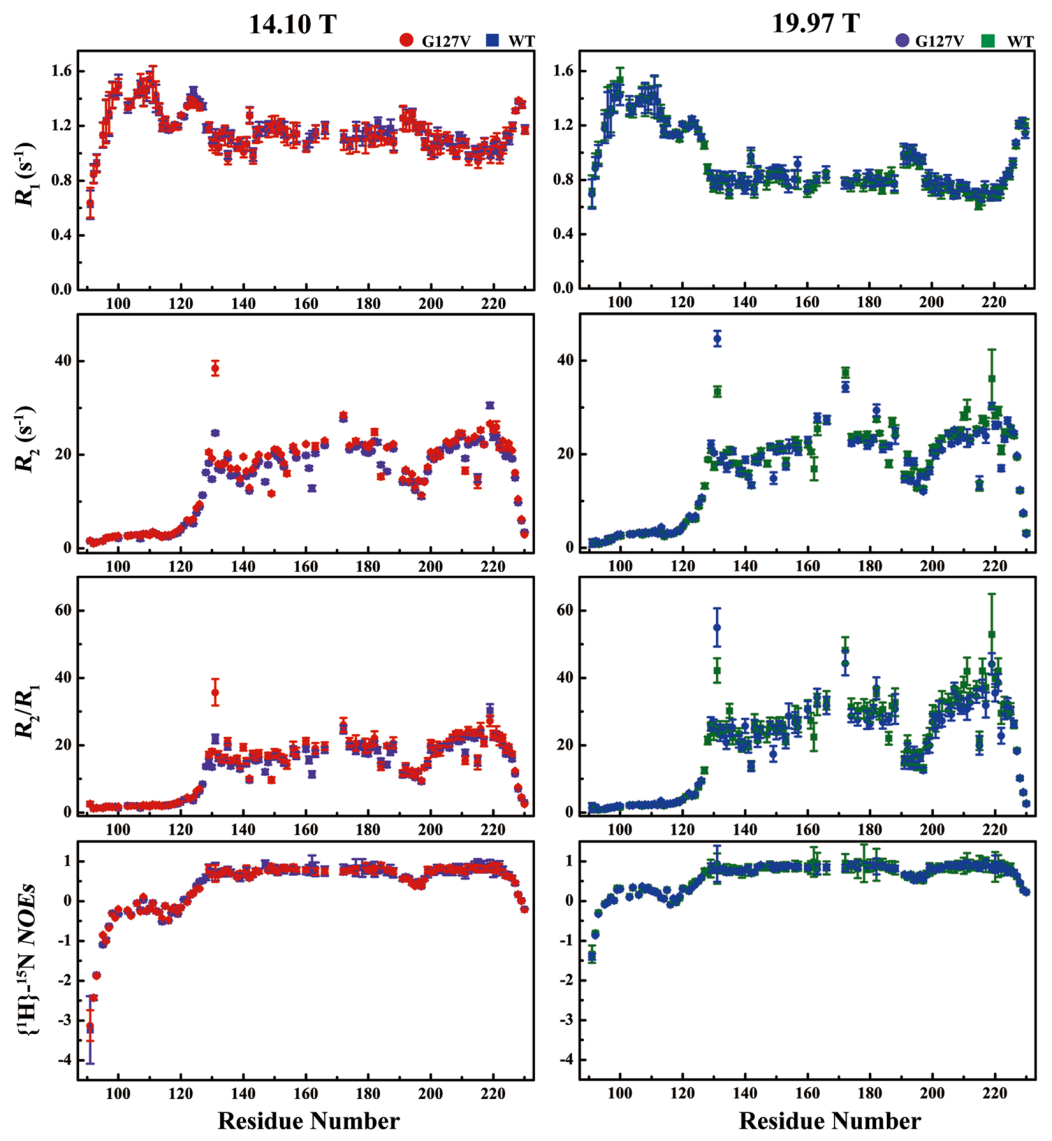
A comparison of the backbone dynamics parameters from HuPrP(G127V) and WT HuPrP derived from ^15^N relaxation data. All NMR spectra were acquired at magnetic field strengths of 14.10 T (red for G127V, violet for WT) and 19.97 T (blue for G127V, olive for WT).

To compare the dynamic features of the HuPrP(G127V) and WT HuPrP backbones, we performed a series of NMR relaxation experiments to obtain ^15^N longitudinal relaxation rates (R_1_), ^15^N transverse relaxation rates (R_2_) and {^1^H}-^15^N heteronuclear steady-state NOEs ({^1^H}-^15^N NOEs) at two magnetic field strengths, 14.10 T and 19.97 T (Fig. 3). A total of 106 and 112 backbone amide resonances were used to analyse the dynamic features of the mutant and WT proteins.

For HuPrP(G127V), residues in the N-terminal flexible segment did not show distinct differences in the R_1_ and R_2_ rates between the two magnetic fields but exhibited more negative {^1^H}-^15^N NOEs at 14.10 T than those at 19.97 T. In contrast, the residues in the C-terminal structural core, except for those in the α2-α3 loop (Gly195-Thr199) and the C-terminus (Gly229-Ser231), displayed significant differences in the R_1_ and R_2_ rates and similar {^1^H}-^15^N NOEs values (>0.6) between the two magnetic fields. The average R_1_ rate at 14.10 T was larger than that at 19.97 T (1.1 s^−1^ vs. 0.8 s^−1^). All residues, except for Gly131 and Gln172 in the C-terminal structural core, displayed R_2_ rates varying between 13.0 s^−1^ and 30 s^−1^ for both magnetic fields, with slightly higher values at 19.97 T. Furthermore, residues in the α2-α3 loop and the C-terminus showed larger R_1_ rates and smaller R_2_ rates as well as smaller {^1^H}-^15^N NOEs. Overall, WT HuPrP showed R_1_ rates, R_2_ rates and {^1^H}-^15^N NOEs roughly similar to HuPrP(G127V) for the two magnetic fields (Fig. 3). Both proteins exhibited larger differences in the R_2_/R_1_ ratios between 14.10 T and 19.97 T.

Interestingly, the G127V mutant showed distinctly changed R_2_ rates for the residues located in the SS1 and SS2 segments. The R_2_ rates of the SS1-located residue Gly131 in the mutant protein were 38.5 s^−1^ at 14.10 T and 44.7 s^−1^ at 19.97 T, which were much larger than those in the WT (24.6 s^−1^ at 14.10 T and 33.4 s^−1^ at 19.97 T). Moreover, Gly131 also displayed significantly different R_2_/R_1_ ratios between the two proteins. Furthermore, the R_2_ rates of the SS2-located residue Tyr163 in the mutant protein were slightly larger than those in the WT (14.10 T: 21.8 s^−1^ vs. 20.3 s^−1^; 19.97 T: 27.9 s^−1^ vs. 25.4 s^−1^). However, the R_2_ rates for the SS1-located residues Met129 and Leu130 in the mutant protein were extremely similar to those in the WT. Regrettably, the relaxation data of the SS2-located residues Val161 and Tyr162 were not suitable for relaxation analysis because of resonance overlapping in the mutant protein.

As expected, the G127V mutation more or less altered the R_2_ rates of the α2 helix residues. Although the R_2_ rate of Gln172 at 14.10 T was almost identical for both the mutant and WT proteins (28.5 s^−1^ vs. 27.6 s^−1^), this value at 19.97 T was smaller in the mutant than that in the WT (34.4 s^−1^ vs. 37.4 s^−1^). Furthermore, Ile182 in the mutant displayed slightly increased R_2_ rates compared to that in the WT protein (24.9 s^−1^ vs. 22.9 s^−1^ at 14.10 T; 29.4 s^−1^ vs. 27.5 s^−1^ at 19.97 T). In addition, the R_2_ rate of Gln186 in the mutant was much larger than that in the WT protein (21.6 s^−1^ vs. 16.4 s^−1^ at 14.10 T; 21.8 s^−1^ vs. 17.9 s^−1^ at 19.97 T). These alterations might be caused by the rotation of the Tyr128 side chain, as described above.

The G127V mutation also changed the R_2_ rates of the residues located within the α3 helix. Because of the retroflexion of the Pro158 pyrrolidine, as described above, the R_2_ rates of Val209 and Val210 subtly fluctuated at 14.10 T and were markedly disturbed at 19.97 T. Compared with the WT protein, the mutant showed slightly larger R_2_ rates for the two residues at 14.10 T (Val209: 24.6 s^−1^ vs. 23.8 s^−1^; Val210: 24.6 s^−1^ vs. 23.6 s^−1^) and displayed smaller R_2_ rates at 19.97 T (Val209: 23.8 s^−1^ vs. 25.1 s^−1^; Val210: 23.5 s^−1^ vs. 28.2 s^−1^) (Fig. 3).

### Reduced spectral density mapping

**Figure 4:**
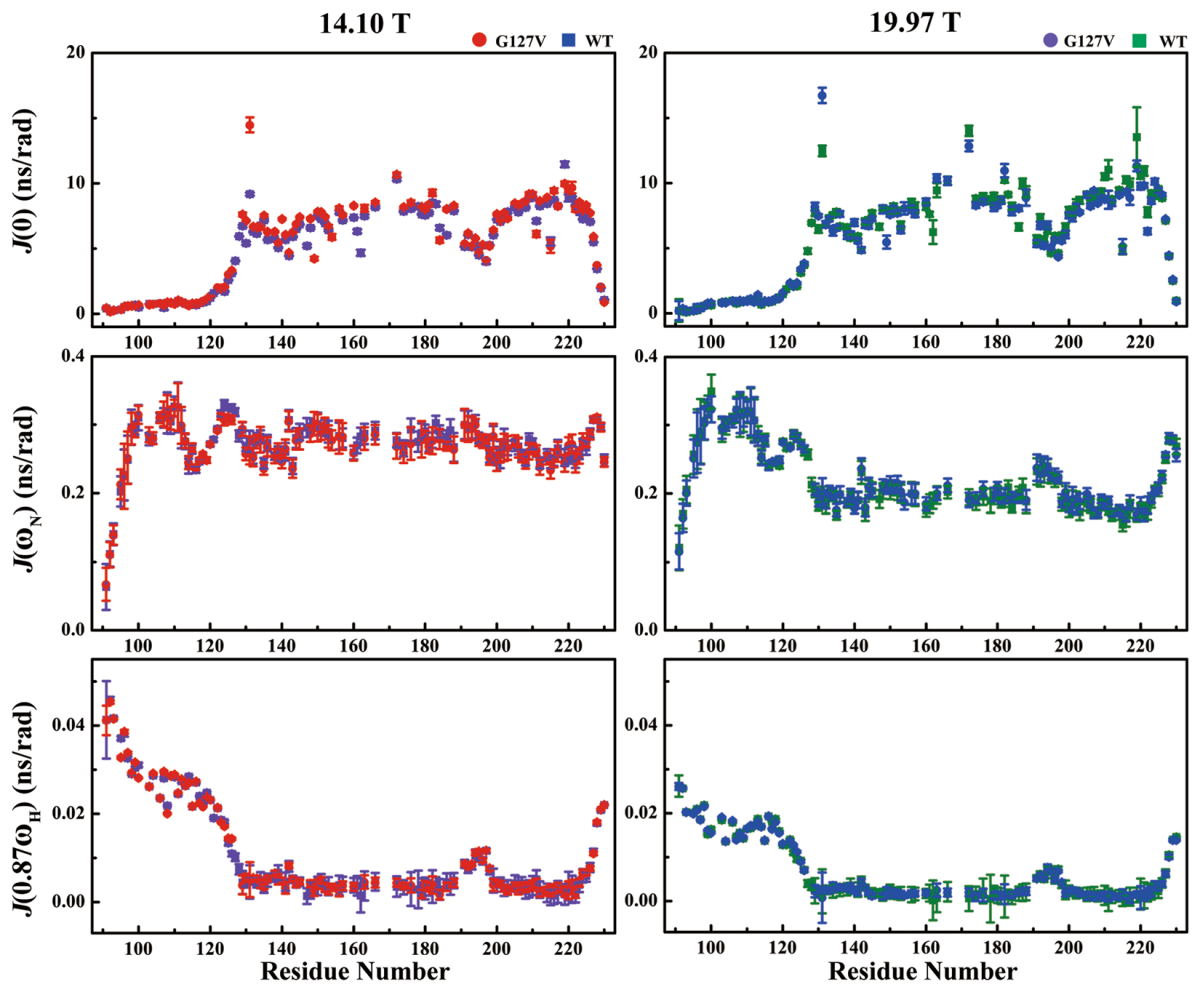
A comparison of the reduced spectral density functions between HuPrP(G127V) and WT HuPrP. All spectral densities were calculated from the corresponding backbone dynamics parameters (R_1_, R_2_, {^1^H}-^15^N NOEs), which were measured at magnetic field strengths of 14.10 T (red for G127V, violet for WT) and 19.97 T (blue for G127V, olive for WT).

To explicitly explore the internal motion of the amide backbone, we calculated the reduced spectral density functions at three frequencies, J(0), J(ω_N_) and J(0.87ω_H_), based on experimentally derived ^15^N relaxation data for both HuPrP(G127V) and WT HuPrP (Fig. 4). For HuPrP(G127V), the J(0) values of the N-terminal flexible segment and the C-terminus were less than 2.5 ns/rad for both magnetic fields. However, the C-terminal structural core displayed J(0) values varying from 5.0 ns/rad to 10.0 ns/rad. The α3 helix exhibited higher J(0) values than the α1 and α2 helices, but the α2-α3 loop displayed relatively smaller J(0) values than the α1 and α2 helices. Moreover, the N-terminal flexible segment showed J(ω_N_) values scattering from 0.05 ns/rad to 0.35 ns/rad at the two magnetic fields, but the C-terminal structural core exhibited J(ω_N_) values fluctuating near 0.27 ± 0.03 ns/rad at 14.10 T and 0.20 ± 0.03 ns/rad at 19.97 T. Furthermore, the N-terminal flexible segment showed J(0.87ω_H_) values between 0.014 ns/rad and 0.045 ns/rad at 14.10 T, which changed to 0.011 ns/rad and 0.026 ns/rad at 19.97 T. The C-terminal structural core displayed J(0.87ω_H_) values varying near 0.006 ns/rad at 14.10 T and 0.003 ns/rad at 19.97 T (Fig. 4). On the whole, compared with HuPrP(G127V), WT HuPrP did not show distinctly different J(0), J(ω_N_), J(0.87ω_H_) values or trends.

Compared with WT HuPrP, HuPrP(G127V) showed much larger J(0) values for the SS1-located residue Gly131 (14.5 ns/rad vs. 9.2 ns/rad at 14.10 T; 16.7 ns/rad vs. 12.5 ns/rad at 19.97 T). The J(0) values for Tyr163 located in the SS2 of the mutant were only subtly larger than those in the WT protein (8.1 ns/rad vs. 7.5 ns/rad at 14.10 T; 10.4 ns/rad vs. 9.5 ns/rad at 19.97 T), similar to the R_2_ rate for Tyr163, which was slightly higher in the mutant than that in the WT protein. These results suggest that the two residues in the mutant underwent slow conformational fluctuations.

Gln172, located in the α2 helix of the mutant protein, displayed almost the same J(0) value as that of the WT protein at 14.10 T (10.7 ns/rad vs. 10.3 ns/rad) but exhibited a smaller J(0) value than that of the WT protein at 19.97 T (12.8 ns/rad vs. 14.0 ns/rad). The anomalous alteration in the J(0) values might have been caused by significant conformational fluctuations. Furthermore, the mutant showed subtly different J(0) values from the WT protein for several residues, including the following: Ile182 (14.10 T: 9.3 ns/rad vs. 8.5 ns/rad; 19.97 T: 11.0 ns/rad vs. 10.2 ns/rad), Gln186 (14.10 T: 8.0 ns/rad vs. 6.0 ns/rad; 19.97 T: 8.1 ns/rad vs. 6.6 ns/rad), Val209 (14.10 T: 9.2 ns/rad vs. 8.8 ns/rad; 19.97 T: 8.9 ns/rad vs. 9.3 ns/rad) and Val210 (14.10 T: 9.2 ns/rad vs. 8.9 ns/rad; 19.97 T: 8.8 ns/rad vs. 10.5 ns/rad). For these residues, the trends in the J(0) values (Fig. 4) were similar to those of the R_2_ rates (Fig. 3).

### Relaxation dispersion measurements

To compare in detail the dynamic features between HuPrP(G127V) and WT HuPrP, especially for Gly131 and Tyr163 with large J(0) values, we performed CPMG RD experiments at pH 4.5 on both the mutant and WT proteins (1.0 mM) at two magnetic field strengths (14.10 T and 19.97 T). The resulting individual interconversion rates (k_ex_) are shown in Table S4.

**Figure 5:**
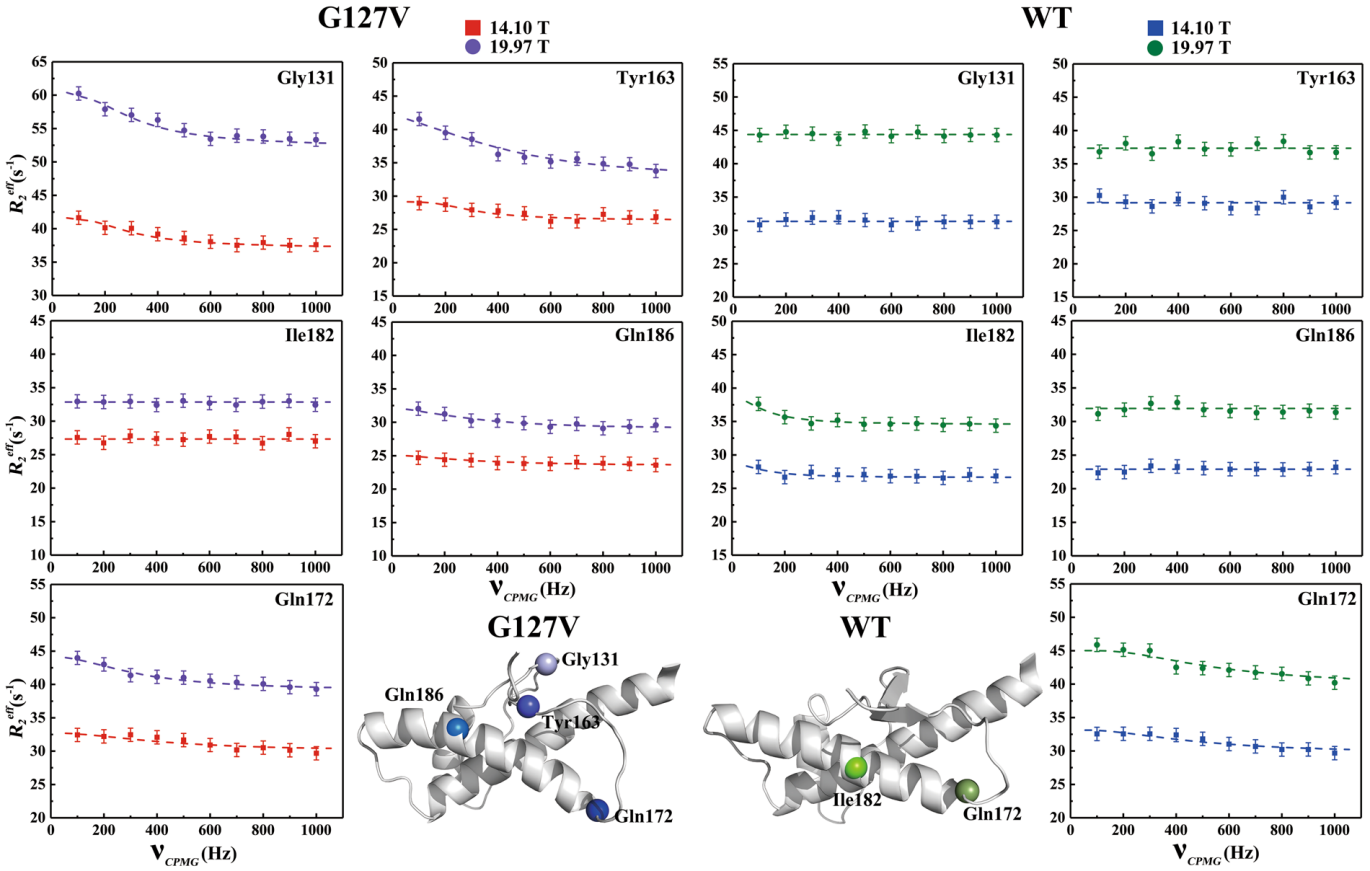
A comparison of μs-ms timescale conformational exchanges between HuPrP(G127V) and WT HuPrP. All CPMG RD experiments were conducted at magnetic field strengths of 14.10 T (red for G127V, violet for WT) and 19.97 T (blue for G127V, olive for WT). The individual interconversion rates (k_ex_) were mapped onto the 3D structures of the mutant and WT proteins. Gly131, Tyr163, Gln172 and Gln186 in the mutant are coloured light blue, TextView blue, blue and marine, respectively. Gln172 and Ile182 from the WT protein are coloured smudge and chartreuse, respectively. The colours of the spheres on the 3D structures show the changing tendency from bright to dark, corresponding to the declining tendency of k_ex_ for the related residues.

For HuPrP(G127V), Gly131 and Tyr163 in the SS1 and SS2 segments displayed k_ex_ rates of 1295 ± 122 s^−1^ and 2842 ± 186 s^−1^, respectively (Fig. 5, Table S4). Notably, residue Tyr128 disappears from the ^1^H-^15^N HSQC spectrum because of peak broadening that may be caused by conformational exchange. The relaxation dispersion data from the Val161 and Tyr162 residues were not suitable for the CPMG RD analysis because of resonance overlapping. Interestingly, Met129 and Leu130 did not display observable conformational fluctuations (Fig. S9, Table S4). Gln172 and Gln186, located in the α2 helix, exhibited significant conformational exchanges, with k_ex_ rates of 3171 ± 302 s^−1^ and 2143 ± 328 s^−1^, respectively (Fig. 5, Table S4).

For WT HuPrP, Gln172 in the nearby the α2 helix displayed conformational exchange with a k_ex_ rate of 1815 ± 281 s^−1^ (Fig. 5; Table S4). Interestingly, Ile182 showed significant conformational exchange (k_ex_ = 1009 ± 486 s^−1^), but Gln186 exhibited negligible conformational exchange (Fig. 5, Table S4), although they are both located in the α2 helix. Furthermore, all residues in the β-sheet displayed insignificant conformational exchanges, excluding the unassigned Arg164 (Fig. S9, Table S4).

Additionally, the α3 helix in both the mutant and WT protein displayed substantial magnetic field strength-dependent conformational fluctuations on the μs-ms timescale (Fig. S10, Table S4). For example, two residues located in the α3 helix, Met205 and Thr216, exhibited significant conformational exchanges at 19.97 T rather than at 14.10 T.

### Molecular dynamics simulations

**Figure 6:**
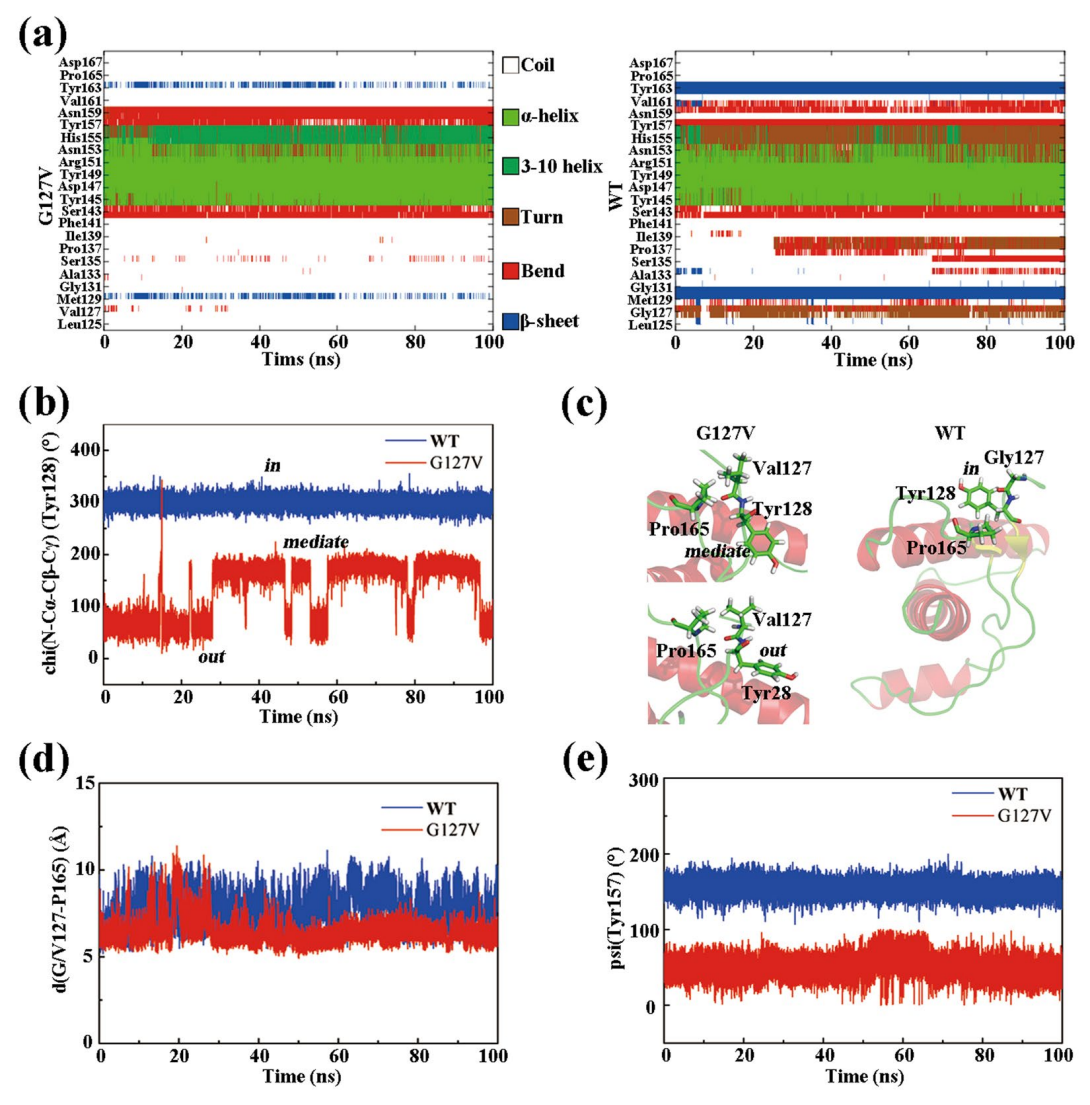
MD simulations of HuPrP(G127V) and WT HuPrP. **(a)** Time evolutions of the secondary structure elements of both proteins. **(b)** Time evolutions of the side chain dihedral angle chi(N-Cα-Cβ-Cγ) of Tyr128. The Tyr128 side chain can adopt three orientations: “out” - pointing to the solvent, “in” - pointing to the protein, and “mediate” - an orientation between “out” and “in”. **(c)** Several unique residues are identified in the 3D structures of the two proteins. **(d)** Time evolutions of the centroid distances between Gly127/Val127 and Pro165. **(e)** Time evolutions of the backbone dihedral angle psi(N-Cα-C-N) of Tyr157.

To further disclose the differences in dynamic structural properties between HuPrP(G127V) and WT HuPrP, we performed MD simulations based on the identified protein structures (Fig. 6 and S11). By analysing secondary structure elements and the geometric relationship of residues Leu125-Asp167, we summarized five primary distinctions between the mutant and WT proteins: the SS1 and SS2 segments rarely form a β-sheet in the mutant, instead, two β-strands always formed a stable β-sheet in the WT during the entire MD simulation (Fig. 6a); the α1 helix in the mutant is extended compared with that in the WT protein (Fig. 6a); the Tyr128 side chain adopts either the “mediate” or “out” conformation in the mutant instead of the “in” conformation found in the WT protein (Fig. 6b,c); the dynamic distance between the mass centres of Val127 and Pro165 in the mutant is smaller than that of Gly127 and Pro165 in the WT protein (Fig. 6d); the dihedral angle psi(N-Cα-C-N) of Tyr157 is approximately 60° for the mutant but is approximately 180° in the WT protein (Fig. 6e). These distinct dynamic properties might derive from the difference in hydrophobicity between Val127 and Gly127. Compared with glycine, valine is more hydrophobic and tends to be near the hydrophobic Pro165 in the SS2-α2 loop, as supported by the 3D structures of the mutant and WT proteins. This spatial alteration might introduce alterations in the conformation of Tyr128 and Tyr157 and the feasibility of β-sheet formation.

More meaningfully, the G127V mutant induces intramolecular steric hindrance in the relatively larger Val127 side chain, leading to a striking structural rearrangement and conformational alternation of the Tyr128 side chain. The MD simulations suggest that the orientation of the Tyr128 side chain directly determined the feasibility of the intermolecular dimerization. For the “exposed” case (either the “mediate” conformation or “out” conformation) in HuPrP(G127V), the steric hindrance closely associated with the Tyr128 side chain potentially prevents the monomeric prion protein from forming intermolecular interactions and might thus prohibit prion dimerization. In contrast, for the “buried” case (the “in” conformation) in WT HuPrP, the Tyr128 side chain likely does not reduce the feasibility of intermolecular dimerization (Fig. 6c). Hence, the mutation-induced structural rearrangement and dramatic conformational exchange of the Tyr128 side chain might be unfavourable for the dimerization and conformational conversion of HuPrP(G127V).

## Discussion

Prion disease pathogenesis is closely associated with the conformational conversion of prion proteins from PrP^C^ to PrP^Sc^. The α2 and α3 helices^33,53,54^, octarepeats^55^, the N-terminal flexible segment^56^, and the glycophosphatidylinositol (GPI) anchor^57^ contained in PrPs might be involved in conformational conversions^58–61^. Moreover, conformational conversion is triggered at the two β-strands, the α1 helix, the α2 helix, the β1-α1 loop, the α1-β2 loop, and the β2-α2 loop^34,35,62–67^. Notably, the more stable β-structure is formed by the segment of the N-terminus (residues 120–144), the earlier stages of misfolding are caused by^43,68,69^ in MD, and a relatively short β-sheet core (residues 112–139) is capable of seeding the conversion to fibrils *in vitro*^70^. Nevertheless, the molecular mechanism underlying the disease-resistant effect of the G127V mutation still remains elusive. To reveal the molecular mechanisms, we determined the solution structures of both the HuPrP(G127V) and WT HuPrP under identical experimental conditions. We then analysed the backbone dynamics using ^15^N relaxation experiments and conducted MD simulations for both proteins. We focused primarily on the dynamic structural properties of the two SS segments and adjacent regions, including intramolecular interactions between SS1 and SS2, SS1/SS2 and α2, SS1/SS2 and α3, α1/α1-SS2 loop and α3, and SS2-α2 loop/α2 and α3.

The primary structural distinction between HuPrP(G127V) and WT HuPrP^22,33^ or other pathogenic mutants^16,23,25–28^ is that HuPrP(G127V) extends atomic distances between SS1 and SS2, increases the solvent accessibility surface of SS1-located residues (Figs S5 and S6), and exhibits significant μs-ms timescale conformational fluctuations at Tyr128, Gly131 and Tyr163. These properties indicate that the SS region is more flexible than the β-sheet and is not prone to conversion to a stable β-sheet conformation. Moreover, the striking structural rearrangement and alternate conformation of the Tyr128 side chain potentially induces the intermolecular steric hindrance effect, prevents the formation of intermolecular hydrogen bonds and prohibits prion protein dimerization. Notably, our result is fundamentally different from a previously published result, which suggested that the intermolecular steric hindrance was closely associated with the bulky sidechain of Val127^71^. The previous MD simulation work was based on the modelled structures of the G127V mutant using the solution structure of WT HuPrP (125–228) determined at pH 7.0 (PDB ID: 1HJN) and the crystal structure of the β1-strand fragment (PDB ID: 4TUT) as the templates^71^. Additionally, HuPrP(G127V) also alters the local electrostatic potential distribution near the SS1 and SS2 segments to influence potentially electrostatic interactions.

Previous studies suggest that pathogenic and protective mutants of PrPs have similar structures and dynamics^29,30,35,36,42,44^. However, our results confirmed that the structural and dynamic alterations caused by G127V are tremendously different from the changes caused by Met129, Val129 or any other known mutants^29,30,35,36,42,44^. Furthermore, as previously hypothesized, the β-sheet in the prion protein, and especially the β1-strand, might be the cornerstone on which prion protein aggregation is triggered^34–36,63,64,66^. For instance, D178N/M129 and F198S form intermolecular antiparallel four-strand β-sheets based on β1-strands in crystal structures^34^, and the β1-strand fragments form a steric zipper conformation^35,36^. However, HuPrP(G127V) possesses flexible SSs with structural rearrangement and conformational fluctuations, rearrangement and alternate conformation of the Tyr128 side chain as well as surface electrostatic potential redistribution that destroys the prion protein aggregation trigger and prohibits prion protein fibrillization.

On the other hand, HuPrP(G127V) and WT HuPrP have similar atomic distances between Met129 and Tyr163 (Table S2), similar H/D exchanges of Met129, Leu130, Val161, Tyr162 and Tyr163 (Figs S7 and S8), and similar dynamic properties for Met129 and Leu130 (Figs 3, 4 and S9). The similar structural and dynamic properties between the SSs in the mutant protein and the β-sheet in the WT protein imply that HuPrP(G127V) might partially reserve the structural and dynamic properties of the β-sheet in the WT protein via the SS pattern.

Regarding the intramolecular interactions between the SSs and the α2/α3 helices in the G127V mutant, we found that (I) the G127V mutant changes the orientation of the Tyr128 side chain and leads to different conformational exchanges for Ile182 and Gln186 (Table S4); (II) the mutation-induced steric hindrance effect between the side chains of Val127 and Arg164 pushes the Arg164 side chain close to Asp178, strengthens the electrostatic interaction between Arg164 and Asp178 (Fig. S2e,f) and enhances the hydrophobic interaction between Val127 and Pro165 (Fig. 6d); (III) the G127V mutant positions two SS2-located residues, Tyr163 and Arg164, slightly closer to Cys179 (Fig. S2g,h). These structural alternations reveal that the G127V mutation changes the local circumstances around the SSs and α2/α3 regions in HuPrP(G127V), which are distinctly different from those in the WT protein and several other HuPrP mutants such as D178N^34^. These unique structural features of HuPrP(G127V) potentially reduce the feasibility of prion protein aggregation^54^.

Furthermore, distinguishing structural features are also identified in the regions around the α1 helix and the α1-SS2 loop and the α2 and α3 helices in HuPrP(G127V), which may be responsible for the prion disease-resistance effects of the G127V mutant. Overall, the G127V mutation extends the α1 helix and induces the retroflexion of the Pro158 pyrrolidine, thus increasing the curvature of the α1-SS2 loop. These structural alterations potentially prevent the unwinding of the α1 helix. As previously suggested, the α1 helix could be converted to the β-strand to form fibrils via a despiralization process^63,64^. In the G127V mutant, the atomic distances between Tyr157, Pro158, Val209 and Val210 changed (Fig. 2g,h) and introduced magnetic field strength-dependent fluctuations in the R_2_ and R_2_/R_1_ rates of Val209 and Val210 (Fig. 3). These alterations might correspondingly change the local environment of the Val210 mutable site (the V210I mutant is associated with *f*CJD^27,49^), and may promote the protective effect of the G127V mutant.

Additionally, the extended α1 helix, the bent α2 helix, and the α3 helix are packed more compactly in HuPrP(G127V) than those in the WT protein. The unique geometric packing in the G127V mutant is similar to the protective packing in the V209M mutant^16^ and might slow the initial fibrillization rate in a manner similar to that in HuPrP(V209M)^16^ and the G126V mutant of the mouse prion protein (moPrP)^72^. The moPrP(G126V) is equivalent to HuPrP(G127V), slows initial fibril growth and increases the critical concentration^72^. The compact geometric packing might also change the local environment of the α2-α3 loop near the α1 helix. Note that the *f*CJD-associated F198S^34^ mutant occurs in the α2-α3 loop. In the G127V mutant, the surface electrostatic potential distribution on the region encompassing the α1 and α3 helices is diametrically distinct from those in the WT and the *f*CJD-associated E200K mutant^26^. The alterations of HuPrP(G127V) in both the geometric packing and electrostatic potential distribution combined with the close atomic distances between SS2 and the disulfide bridge, might prohibit rearrangement of the disulfide bridge, aggregation and fibrillization as previously published results^16,33,54,72^.

Compared with the WT HuPrP, the SS2-α2 loop (Pro165-Asn171) of the HuPrP(G127V) exhibits more flexibility. The G127V mutation allows Met166 at the SS2-α2 loop to be closer to Tyr218, which is located in the α3 helix (Fig. S2i,j). In HuPrP(G127V), Gln172, next to the SS2-α2 loop, undergoes a more significant conformational exchange than that in the WT HuPrP (Table S4). These results indicate that the SS2-α2 loop has dynamic structural features distinct from the β2-α2 loop (Pro165-Gln172), which is probably correlated to the susceptibility to prion disease^65^. The unique dynamic structural properties of the SS2-α2 loop might contribute to the prion disease resistance of the G127V mutant as well.

Astonishingly, the α3 helix in HuPrP(G127V) showed R_2_/R_1_ ratios that were dramatically different from those of the WT HuPrP at 19.97 T (Fig. 3). Moreover, the α3 helix exhibited varying J(0) values, similar to the R_2_/R_1_ ratios. In addition, Met205 and Thr216 in both proteins experienced slow conformational exchange, which was observable only at 19.97 T. Unexpectedly, the α3-located Glu219 in both proteins displayed large R_2_ rates and J(0) values but did not exhibit observable conformational exchanges (Fig. S10, Table S4). Furthermore, Glu219 in HuPrP(G127V) showed the dynamic property, distinct from HuPrP(E219K)^23^. Thus, the dramatically altered dynamic structural properties relevant to the α3 helix could potentially influence the intermolecular interactions of the prion protein with the so called “protein X”^73,74^.

Besides, our fibrillization experiments showed that HuPrP(G127V) had significantly slower initial fibril growth than WT HuPrP. The measured lag phases were 61 ± 2 h for HuPrP(G127V) and 25 ± 2 h for WT HuPrP as showed in Fig. S12. Moreover, the mixing samples of WT HuPrP and HuPrP(G127V) (at a mixing ratio of 1:1) exhibited a slower fibrillization rate than WT HuPrP but faster than HuPrP(G127V). The measured lag phase was 47 ± 2 h for the mixing sample. These kinetic analyses are similar to the quantitative comparison of moPrP(G126V) and WT moPrP^72^. These unique dynamic structural features might be responsible for the prion disease-resistance effect of the G127V mutant^20,21^. As expected, the further study of the exploitation of the structural and dynamic features of the GSS-associated mutant G131V^75,76^ (GSS), which was confirmed to enhance the stability of the β-sheet and drive conformational conversion by MD simulation^77,78^, would greatly help to address the crucial role of the SS1 segment in conformational conversion and propagation.

Summarily, we performed solution structure determinations, NMR dynamics analysis and MD simulations on both HuPrP(G127V) and WT HuPrP. We addressed the G127V mutation-induced significant distinct alterations in structural and dynamic properties in detail. The G127V mutation extends atomic distances between the SS1 and SS2 segments and enhances the conformational exchange of the two strands, leading to the formation of the SS pattern instead of the stable β-sheet. The relatively larger hydrophobic side chain of Val127 introduces steric hindrance and a striking structural rearrangement in the Tyr128 side chain. Additionally, the G127V mutation also subtly alters the geometric stacking of the three α helices. These structural and dynamic features might prevent the SS1 (Tyr128-Gly131) and SS2 (Val161-Arg164) segments and adjacent regions from being converted into a stable β-sheet under certain circumstances. Furthermore, the steric hindrance effect of the rearrangement of the Tyr128 side chain, together with the dramatic conformational alternation, could potentially prohibit the prion protein intermolecular interaction and dimerization, and thus inhibit prion protein aggregation and fibrillization. Moreover, HuPrP(G127V) had significantly slower initial fibril growth than WT HuPrP. Although more researches are required to clarify completely the molecular mechanisms of the prion disease-resistance of HuPrP(G127V), our results provide several important evidences regarding the differences in structure and dynamics between HuPrP(G127V) and WT HuPrP. These structural and dynamic differences substantially contribute to the different conversion of monomer to dimer in MD and of monomer to fibril in fibrillization between the two proteins. This work may be helpful for mechanistically understanding the pathogenesis of prion diseases and for developing effective drugs against prion diseases.

## Methods

### NMR sample preparation

Recombination of the pET30a plasmids without any tag bearing the DNA of the WT HuPrP (residues 91–231 with G127M129) was prepared as previously described^79–81^. The recombination plasmids for HuPrP(G127V) (residues 91–231 with the genotype of V127M129) were cloned by PCR using site-directed mutagenesis. The forward primer used in the PCR was: 5′-AGTGGTGGGGGGCCTTGGCGTTTACATGCTGGGAA-3′ and the reverse primer used was: 5′-ATGGCACTTCCCAGCATGTAAACGCCAAGGCCCCCCA-3′. The uniformly labelled protein was overexpressed in *E*. *coli* Bl21(DE3) grown in M9 medium. ^15^NH_4_Cl was added to the M9 medium to prepare ^15^N-labelled proteins and both ^15^NH_4_Cl and ^13^C_6_-glucose were added to prepare ^13^C/^15^N-labelled proteins. After the cells were sonicated and the lysates were centrifuged, the inclusion bodies were denatured in 6 M guanidine hydrochloride and refolded by dialysing against the NMR buffer (20 mM NaOAc, 0.02% NaN_3_, pH 4.5) as previously described^79–81^. Thereafter, the protein was purified in NMR buffer through size exclusion chromatography with Superdex-75 on an ÄKTA FPLC system (GE Healthcare). Finally, the protein solution was concentrated to approximately 0.5 mM with 10% D_2_O (v/v).

### NMR spectroscopy

To perform backbone and side chain resonances and to determine the solution structures of the HuPrP(G127V) and WT HuPrP proteins, we recorded a suite of 2D/3D heteronuclear NMR spectra at 25 °C on a Bruker Avance III 850-MHz spectrometer (magnetic field strength is 19.97 T) with a ^1^H/^13^C/^15^N triple-resonance cryogenic probe (TCI). These 3D NMR spectra included HNCACB, CBCA(CO)NH, HNCA, HNCOCA, HNCO, HN(CA)CO, HBHA(CO)NH, H(CCCO)NH, CC(CO)NH and (H)CCH-TOCSY. A mixing time of 120 ms was used for both ^15^N-edited NOSEY-HSQC and ^13^C-edited NOESY-HSQC experiments. All NMR spectra were processed by NMRPipe software^82^ and analysed with CARA software^83^.

### Structure calculations

Distance constraints were generated from the ^1^H-^1^H NOEs of both ^13^C and ^15^N-labelled NOESY-HSQC spectra. Dihedral angle restraints were obtained based on chemical shifts of the backbone atoms including HN, Hα, Cα, Cβ, C(O), and N using the TALOS+ programme^84^. The 3D structures were calculated and refined with the XPLOR-NIH package^85^. Then, the qualities of the calculated structures were evaluated by the PROCHECK programme^86^. Ultimately, the 20 lowest-energy conformers were selected as representative solution structures of each protein. The tertiary structures of the proteins were displayed using PyMOL^87^ and MOLMOL^88^.

### Backbone dynamics

#### Backbone amide relaxation measurements

A complete set of backbone amide R_1_, R_2_ and {^1^H}-^15^N NOEs spectra were acquired on ^15^N labelled samples on both a Bruker Avance III 850-MHz (magnetic field strength of 19.97 T with a TCI cryogenic probe) and a Bruker Avance III 600-MHz (magnetic field strength of 14.10 T with a BBO cryogenic probe) spectrometer at 25 °C and pH 4.5. All spectra were recorded with 1024 × 128 complex points. R_1_ values were measured from 2D ^1^H-^1^N HSQC spectra with relaxation delays of 10, 50, 100 (×2), 250, 500, 800, 1200 (×2), 1600 and 2000 ms. R_2_ values were determined with relaxation delays of 16.32, 32.64 (×2), 48.96, 65.28, 81.6, 97.92, 114.24(×2), 130.56, 146.88, and 163.2 ms. The repeated spectra were used for experimental error analysis. {^1^H}-^15^N NOEs were obtained by recording spectra with a ^1^H pre-saturation of 3 s plus a 2-s relaxation delay and without a pre-saturation of a 5-s relaxation delay. All NMR spectra were processed using NMRPipe software^82^ and analysed using CcpNmr software^89^. Peak heights were used to represent peak intensities. Standard errors of the fitted parameters were obtained by Monte Carlo simulations. For HuPrP(G127V), 21 residues were unavailable for NMR dynamics analysis due to resonance overlapping: 101, 121, 127, 146, 147, 155, 159, 161, 162, 173, 177, 178, 183, 185, 187, 189, 190, 204, 213, 218 and 231. Thus, 106 backbone amide resonances were used to analyse the dynamic behaviour of the molecular backbone. For WT HuPrP, 16 residues could not be used for NMR dynamics analysis due to resonance overlapping: 101, 140, 146, 149, 154, 155, 159, 173, 177, 185, 189, 190, 204, 213, 218, and 231. Thus, 112 backbone amide resonances were employed to analyse the dynamic behaviour of the molecular backbone.

### Reduced spectral density mapping

Reduced spectral density mapping is usually employed to characterize the internal motions of the N-H bonds with the assumption that *J*(0.87*ω*_*H*_) is approaching *J*(*ω*_*H*_ + *ω*_*N*_) and *J*(*ω*_*H*_ + *ω*_*N*_) at high frequencies^90^. Therefore, the values of the relaxation rates R_1_, R_2_ and {^1^H}-^15^N NOEs are taken to map the spectral density using the following formula:

1 $${\sigma }_{NH}=\,{R}_{1}(NOE-1){\gamma }_{N}/{\gamma }_{H}$$

2 $$J(0)=(6{R}_{2}-3{R}_{1}-2.72{\sigma }_{NH})/(3{d}^{2}+4{c}^{2})$$

3 $$J({\omega }_{N})=(4{R}_{1}-5{\sigma }_{NH})/(3{d}^{2}+4{c}^{2})$$

4 $$J(0.87{\omega }_{H})=4{\sigma }_{NH}/(5{d}^{2})$$

where

$$d=\,{\mu }_{0}h{\gamma }_{N}{\gamma }_{H}({{r}_{NH}}^{-3})/(8{\pi }^{2})$$

and

$$c=\,{\omega }_{N}\Delta \sigma /\sqrt{3}$$

where *μ*_0_ is the permeability of the free space; *h* is Planck’s constant; *γ*_*N*_ and *γ*_*H*_ are the gyromagnetic ratios of ^15^N and ^1^H, respectively; *ω*_*N*_ and *ω*_*H*_ are the Larmor frequencies of ^15^N and ^1^H, respectively; *r*_*NH*_ is the length of the N–H bond; and ${\Delta }\sigma$ = ${\sigma }_{\parallel }-{\sigma }_{\perp }$ is the chemical shift anisotropy for ^15^N. The calculations were implemented using Mathematica software^91^.

### Relaxation dispersion measurements

Single quantum CPMG RD experiments were performed on the same NMR instruments described above (850 MHz at 19.97 T with a TCI cryogenic probe, 600 MHz at 14.10 T with a BBO cryogenic probe). The CPMG RD spectra were recorded on ^15^N-edited HuPrP(G127V) and WT HuPrP proteins at 25 °C and pH 4.5 using a constant relaxation time of 40 ms and under thirteen *ν*_*CPMG*_ values of 0, 100(×2), 200, 300, 400, 500, 600, 700(×2), 800, 900, and 1000 Hz. All spectra were recorded with complex points of 1024 × 128. The *ν*_*CPMG*_ is defined by the following formula^92^:

5 $${\nu }_{CPMG}=\,\frac{1}{4{\tau }_{cp}}$$

Here, *τ*_*cp*_ is the time between refocusing pulses during the CPMG pulse train. We used the following equation^92^ to calculate the effective transverse relaxation rates, ${R}_{2}^{eff}$

6 $${R}_{2}^{eff}({\nu }_{CPMG})\,=\,\frac{-1}{{T}_{cp}}\,\mathrm{ln}\,\frac{I({\nu }_{CPMG})}{{I}_{0}}$$

where *T*_*cp*_ is the constant transverse relaxation time and *I*(*ν*_*CPMG*_) and *I*_*0*_ are the intensity with or without different *ν*_*CPMG*_. The RD of ${R}_{2}^{eff}$ relies on *ν*_*CPMG*_ if the residue undergoes conformational exchange at the μs-ms timescale. All spectra were processed in NMRPipe^82^ and the integrals of the peaks were obtained in NMRFAM-Sparky^93^. The dispersion data were fitted with a Carver-Richards two-state exchange model^94^ in NESSY software^95^. Similar to the ^15^N backbone dynamics analysis, overlapping amide resonances were not used for CPMG RD analysis.

### Molecular Dynamics Simulations

All MD simulations were performed with the AMBER99SB^96^ force field in AMBER12^97^. All systems were solvated within a cubic box of TIP3P^98^ water molecules by extending 10 Å from the protein surface. The initial coordinates and topology files were generated using the tleap programme contained in AMBER12. First, energy minimizations were performed to relax the solvent and optimize the system. Then, each system was gradually heated from 0 to 300 K under the NVT ensemble for 100 ps and another 100 ps of NPT ensemble MD simulation was performed at 300 K and a target pressure of 1.0 atm. Finally, a 100 ns MD simulation under the NVT ensemble was performed for each model. The system temperature was controlled by the Langevin thermostat method. During the MD simulations, all hydrogen-containing bonds were constrained using the SHAKE algorithm^99^. A cut-off of 12 Å was set for both the van der Waals and electrostatic interactions. The DSSP algorithm was employed to assign the secondary structure of the protein^100^.

### Residual Dipolar Couplings

Initially, both ^15^N-labeled HuPrP(G127V) and WT HuPrP were dissolved in H_2_O buffer (90% H_2_O, 10% D_2_O, 20 mM NaOAc, 0.02% NaN_3_, pH 4.5) to a final concentration of 0.4 mM. As reference spectra, 2D ^1^H-^15^N IPAP-HSQC spectra were recorded at 25 °C on a Bruker Avance III 600-MHz spectrometer (magnetic field strength of 14.10 T with a triple-resonance TCI cryogenic probe) at the University of Science and Technology of China. All spectra were recorded with complex points of 1024 × 400. Then, the two proteins were dissolved in C_12_E_5_/n-hexanol alignment media^101^. The final concentration of C_12_E_5_ was 3% (r = 0.96)^101^. 2D ^1^H-^15^N IPAP-HSQC spectra were recorded under the same experimental conditions. All data were processed on NMRPipe^82^, analysed on NMRFAM-Sparky^93^ and fitted on PALES^102^. The Q-value was fitted by PALES, which is normally used to assess the agreement between the experimental RDCs and calculated RDCs based on the structure^102,103^. When fitted using the PALES program, the experimental RDCs were just from the residues of the C-terminal structural core minus the overlapping resonance, as described above.

### Amide Hydrogen/Deuterium Exchange

Both ^15^N labelled HuPrP(G127V) and WT HuPrP were initially dissolved in H_2_O buffer (90% H_2_O, 10% D_2_O, 20 mM NaOAc, 0.02% NaN_3_, pH 4.5). As reference spectra, 2D Fast-^1^H-^15^N HSQC^104^ were recorded at 25 °C on a Bruker Avance III 850-MHz spectrometer (magnetic field strength of 19.97 T with a triple-resonance TCI cryogenic probe). All spectra were recorded with complex points of 1024 × 128. Through buffer exchange with centrifugal filter devices (Amicon® Ultra 3 K device) at 2,555 × g and 4 °C for 3 h, the proteins were re-dissolved in equal volumes of D_2_O buffer (99.9% D_2_O, 20 mM NaOAc, 0.02% NaN_3_, pH 4.5). Then, 2D Fast-^1^H-^15^N HSQC spectra were recorded on the re-dissolved proteins as amide proton exchange spectra under the same experimental conditions. All data were processed on Topspin 3.2 (Bruker) and analysed in CcpNmr^89^. This approach allowed the quantitative analysis of peak intensity decreases caused by the mutation but could not be used to measure amide protection factors for the protein^105^.

### Accession codes

Chemical shift data were deposited in the Biological Magnetic Resonance Data Bank (http://www.bmrb.wisc.edu) under accession numbers 27259 for HuPrP(G127V) and 27264 for WT HuPrP. The atomic coordinates were deposited in the Protein Data Bank under the accession codes 5YJ4 for HuPrP(G127V) and 5YJ5 for WT HuPrP.

## Electronic supplementary material

Supplementary information
