# Supplementary material for: Structural basis for the complete resistance of the human prion protein mutant G127V to prion disease
Source: Sci Rep. 2018 Sep 4;8:13211. doi: 10.1038/s41598-018-31394-6 (PMC6123418; doi:10.1038/s41598-018-31394-6)
Supplement: Supplementary file 1 — Supplementary information [file 41598_2018_31394_MOESM1_ESM.pdf]

Supplementary information

Structural basis for the complete resistance of the human prion protein mutant G127V to prion disease

Zhen Zheng[1], Meilan Zhang[1], Yongheng Wang[2], Rongsheng Ma[3], Chenyun Guo[1], Liubin Feng[1], Jihui Wu[3], Hongwei Yao[1], Donghai Lin[1]*

1. MOE Key Laboratory of Spectrochemical Analysis & Instrumentation, Key Laboratory of Chemical Biology of Fujian Province, College of Chemistry and Chemical Engineering, Xiamen University, Xiamen, 361005, China
2. School of Pharmaceutical Sciences, Sun Yat-sen University, Guangzhou, 510006, China
3. School of Life Sciences, University of Science and Technology of China, Hefei, 230026, China

*Correspondence address: Tel/Fax: +86-592-2186078; E-mail: dhlin@xmu.edu.cn

Table S1. Statistics for 3D structures of HuPrP(G127V) and WT HuPrP.

	HuPrP(G127V) PDB ID 5YJ4	WT HuPrP PDB ID 5YJ5**
Distance restraints		
NOE upper distance restraints	2320	2421
Intra-residue (| i – j | = 0)	1025	1304
Sequential (| i – j | = 1)	762	640
Medium range (1 < | i – j | < 5)	198	221
Long range (| i – j | ≥ 5)	335	256
Dihedral angle restraints (Φ and Ψ)	168	174
Number of restraint violations		
Distance violations (> 0.3 Å)	0	0
Dihedral angle violations (> 5°)	0	0
RMSD from mean structure (Å)		
Backbone atoms (residues 125-228)*	1.28 ± 0.27	0.99 ± 0.21
Heavy atoms (residues 125-228)	2.03 ± 0.23	1.76 ± 0.20
Secondary structure backbone atoms	0.57 ± 0.14	0.64 ± 0.14
Secondary structure heavy atoms	1.35 ± 0.14	1.37 ± 0.16
Ramachandran statistics (residue 125-231)		
Most favoured regions (%)	82.3	84.9
Additional allowed regions (%)	15.6	12.8
Generously allowed regions (%)	2.2	2.3
Disallowed regions (%)	0	0

Note: "*" Because of the absence of the anti-parallel β sheet formed between residues 128-131 and 161-164 and the lack of NOE restraints for residues 165-171, the backbone RMSD of the mutant is somewhat larger than that of the WT protein. The structural differences were verified by MD simulations conducted on the determined 3D structures of the two proteins (Figure S11).

"**" The current solution structure of WT HuPrP is strikingly similar to the previously reported solution structure (PDB ID: 1QM0) with a backbone RMSD of 1.47 Å between the two average structures.

Table S2. Backbone atomic distances between SS1 and SS2 in HuPrP(G127V) and those between the β1-strand and the β2-strand in WT HuPrP.

		HuPrP(G127V) (Å)	WT HuPrP (Å)
Potential backbone hydrogen bonds (1.3 Å - 2.3 Å)			
HN_{Met129}-O_{Tyr163}		-	2.0
O_{Met129}-HN_{Tyr163}		2.3	1.7
H_{SS1}-H_{SS2} or $H_{\beta 1}$-$H_{\beta 2}$ distances (≤ 5.0 Å)			
$H\alpha_{Tyr128}$	$H\alpha_{Arg164}$	-	4.1
	HN_{Arg164}	-	4.5
	$H\alpha_{Tyr163}$	4.4	3.4
	HN_{Tyr163}	3.8	3.4
	$H\alpha_{Tyr162}$	4.2	-
HN_{Met129}	$H\alpha_{Arg164}$	4.2	3.5
	HN_{Arg164}	-	4.7
	$H\alpha_{Tyr163}$	4.3	4.1
	HN_{Tyr163}	2.6	2.6
	$H\alpha_{Tyr162}$	3.4	4.5
$H\alpha_{Met129}$	HN_{Tyr163}	4.9	4.2
	$H\alpha_{Tyr162}$	5.0	4.6
HN_{Leu130}	HN_{Tyr163}	-	4.7
	$H\alpha_{Tyr162}$	-	4.2
$H\alpha_{Leu130}$	HN_{Tyr163}	3.8	3.5
	$H\alpha_{Tyr162}$	3.7	2.2
	HN_{Tyr162}	-	4.7
	HN_{Val161}	4.6	4.2
HN_{Gly131}	$H\alpha_{Tyr162}$	-	3.9
	HN_{Val161}	4.9	3.7

Note: "-" denotes that the distance is beyond the preset range.

Table S3. A comparison of primary differential ^{1}H-^{1}H NOE peak intensities and corresponding ^{1}H-^{1}H distances measured from determined solution structures between the SS region of HuPrP(G127V) and the β-sheet of WT HuPrP.

		Differential NOE comparison (relative intensities)		Averaged distance comparison (Å)	
		HuPrP(G127V)	WT HuPrP	HuPrP(G127V)	WT HuPrP
^{15}N-edited NOESY-HSQC spectra					
HN_{M129}	HA_{Y162}	0.39	0.26	3.4	4.5
	HA_{Y163}	0.19	0.34	4.3	4.1
	HD_{Y162}	0.82	0.59	2.5	4.8
HN_{G131}	HA_{Y162}	0.05	0.52	-	3.9
	HN_{V161}	0.27	0.67	4.9	3.7
	HE_{Y163}	0.38	0.85	3.6	1.9
	HD_{Y163}	○	0.58	-	4.0
HN_{V161}	$HD2_{L130}$	0.63	0.83	3.2	2.0
HN_{Y163}	$HD2_{L130}$	0.28	0.37	-	4.7
	HA_{L130}	0.65	0.82	3.8	3.5
	HN_{M129}	1.00*	1.00*	2.6	2.6
	$HG2_{M129}$	0.20	0.41	4.6	3.4
^{13}C-edited NOESY-HSQC spectra					
HB_{Y128}	HD_{Y162}	0.29	0.40	4.2	3.9
$HD2_{L130}$	HN_{V161}	0.17	0.39	4.7	4.0
	HA_{Y162}	0.08	0.31	-	4.1
$HA2_{G131}$	HE_{Y163}	0.28	0.30	4.5	4.2
HA_{Y162}	$HD2_{L130}$	0.19	0.50	4.3	2.4
	HG_{L130}	0.17	0.33	4.2	4.1
	$HB3_{L130}$	0.05	0.57	-	3.4
	$HB2_{L130}$	0.19	0.25	-	4.7
	HA_{L130}	0.36	0.85	3.7	2.2
	$HB2_{Y128}$	0.19	0.26	4.7	4.5
$HA3_{G131}$	$HA2_{G131}$	1.00*	1.00*	1.8	1.8

Note: "○" represents the peak is invisible in the ^{1}H-^{15}N-edited NOESY-HSQC spectra;

" - " denotes that the distance is beyond the preset range (5.0 Å).

" * " denotes that the intensities are the basis of normalization in ^{15}N-edited NOESY-HSQC and ^{13}C-edited NOESY-HSQC.

Table S4. Individual interconversion rates (k_{ex}) of residues located in secondary structure elements of HuPrP(G127V) and WT HuPrP measured via CPMG RD experiments.

Secondary structure	Residue	HuPrP(G127V) (s^{-1})	WT HuPrP (s^{-1})
	Val127/Gly127	●	-
SS1 / β1	Tyr128	○	-
	Met129	-	-
	Leu130	-	-
	Gly131	1295 ± 122	-
SS2 / β2	Val161	●	-
	Tyr162	●	-
	Tyr163	2842 ± 186	-
	Arg164	○	○
α2 / β2-α2 loop	Gln172	3171 ± 302	1815 ± 281
α2	Ile182	-	1009 ± 486
	Gln186	2143 ± 328	-
α3	Met205*	408 ± 231	491 ± 217
	Thr216*	806 ± 266	347 ± 219
	Glu219	-	-

Note: "●" represents residues with overlapped peaks in either ^{1}H-^{15}N HSQC spectra or CPMG RD spectra;

"○" represents residues invisible in the ^{1}H-^{15}N HSQC spectra;

"-" represents residues with negligible interconversion rates.

"*" indicates k_{ex} values measured only at 19.97 T.

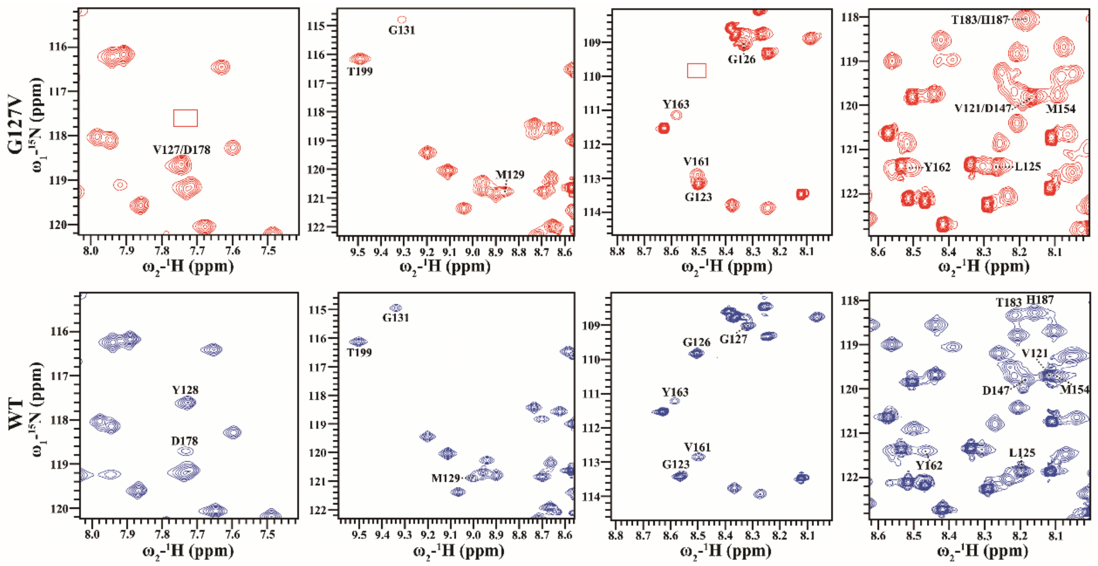

Figure S1. A comparison of 2D ^{1}H-^{15}N HSQC spectra for HuPrP(G127V) (red) and WT HuPrP (blue). All peaks with distinct differences shown in rectangular frames are associated with the two segments (residues 127-131, 161-163) and the residues adjacent to the two segments in either the sequence or the space. The peak differences between the two proteins might be related to dynamic structural alterations caused by the G127V mutation. The spectra were recorded at a magnetic field strength of 19.97 T.

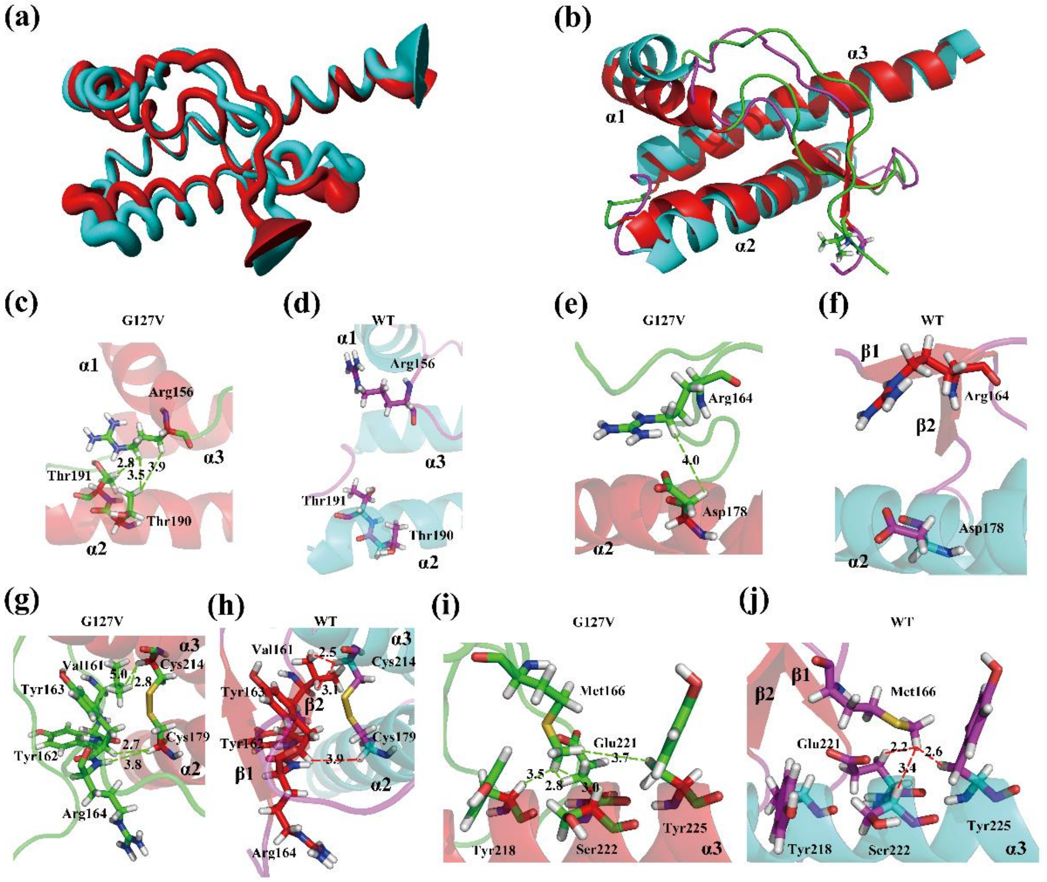

Figure S2. A comparison of the 3D structures of HuPrP(G127V) and WT HuPrP. (a) Overlapping sausages of the structural ensembles of the mutant (red) and WT (cyan) proteins. (b) Overlapping cartoons of the average structures of both proteins with the same colour pattern as in panel a. A backbone RMSD of 2.27 Å was calculated between the two average structures. (c) The mutant protein shows short atomic distances between helix α1 and helix α2 ($d_{H\beta/Arg156\text{-}dH\gamma/Thr190}$, $d_{H\delta/Arg156\text{-}H\gamma/Thr190}$, $d_{H\delta/Arg156\text{-}H\beta/Thr191}$ < 5.0 Å). (d) The WT protein does not show these short atomic distances between helix α1 and helix α2. (e) The mutant protein shows short atomic distances between Arg164 and Asp178 ($d_{H\delta/Arg164\text{-}H\beta/Asp178}$ < 5.0 Å). (f) The WT protein does not show short atomic distances between Arg164 and Asp178. (g) The mutant protein shows short atomic distances between SS2 and the disulfide bridge ($d_{H\beta/Arg161\text{-}H\alpha/Cys214}$, $d_{H\gamma/Arg161\text{-}H\alpha/Cys214}$, $d_{H\alpha/Tyr163\text{-}H\alpha/Cys179}$, $d_{HN/Arg164\text{-}H\alpha/Cys179}$ < 5.0 Å). (h) The WT protein shows short atomic distances between the β2-strand and the disulfide bridge ($d_{H\beta/Arg161\text{-}H\alpha/Cys214}$, $d_{H\gamma/Arg161\text{-}H\alpha/Cys214}$, $d_{H\alpha/Tyr163\text{-}H\alpha/Cys179}$ < 5.0 Å). (i) The mutant shows short atomic distances between the SS2-α2 loop and the α3 helix ($d_{H\varepsilon/Met166\text{-}H\alpha/Tyr218}$, $d_{H\varepsilon/Met166\text{-}H\beta/Glu221}$, $d_{H\varepsilon/Met166\text{-}H\alpha/Ser222}$, $d_{H\varepsilon/Met166\text{-}H\beta/Tyr225}$ < 5.0 Å). (j) The WT protein shows short atomic distances between the β2-α2 loop and the α3 helix ($d_{H\varepsilon/Met166\text{-}H\beta/Glu221}$, $d_{H\varepsilon/Met166\text{-}H\alpha/Ser222}$, $d_{H\varepsilon/Met166\text{-}H\beta/Tyr225}$ < 5.0 Å).

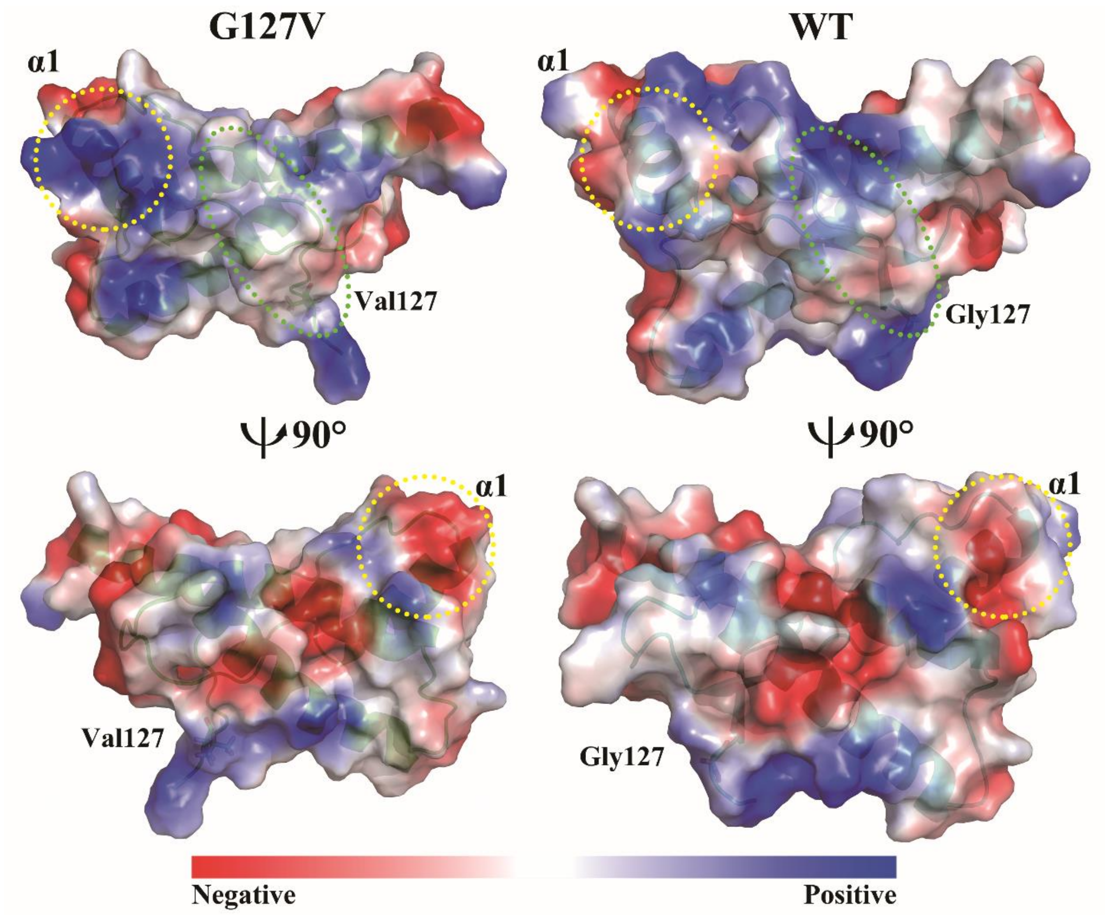

Figure S3. Surface electrostatic potential distributions of 3D structures calculated for HuPrP(G127V) and WT HuPrP. The region near residues Gly126-Ser135 in the mutant displays a neutral potential distribution labelled with an olive-coloured dotted ellipse. Remarkably, the corresponding region in the WT protein shows a positive potential distribution except for near residues Met129, Leu130 and Gly131. Notably, the surface electrostatic potentials around the α1 helix are redistributed once Gly127 is replaced by Val127, which is marked by a yellow-coloured dotted ellipse.

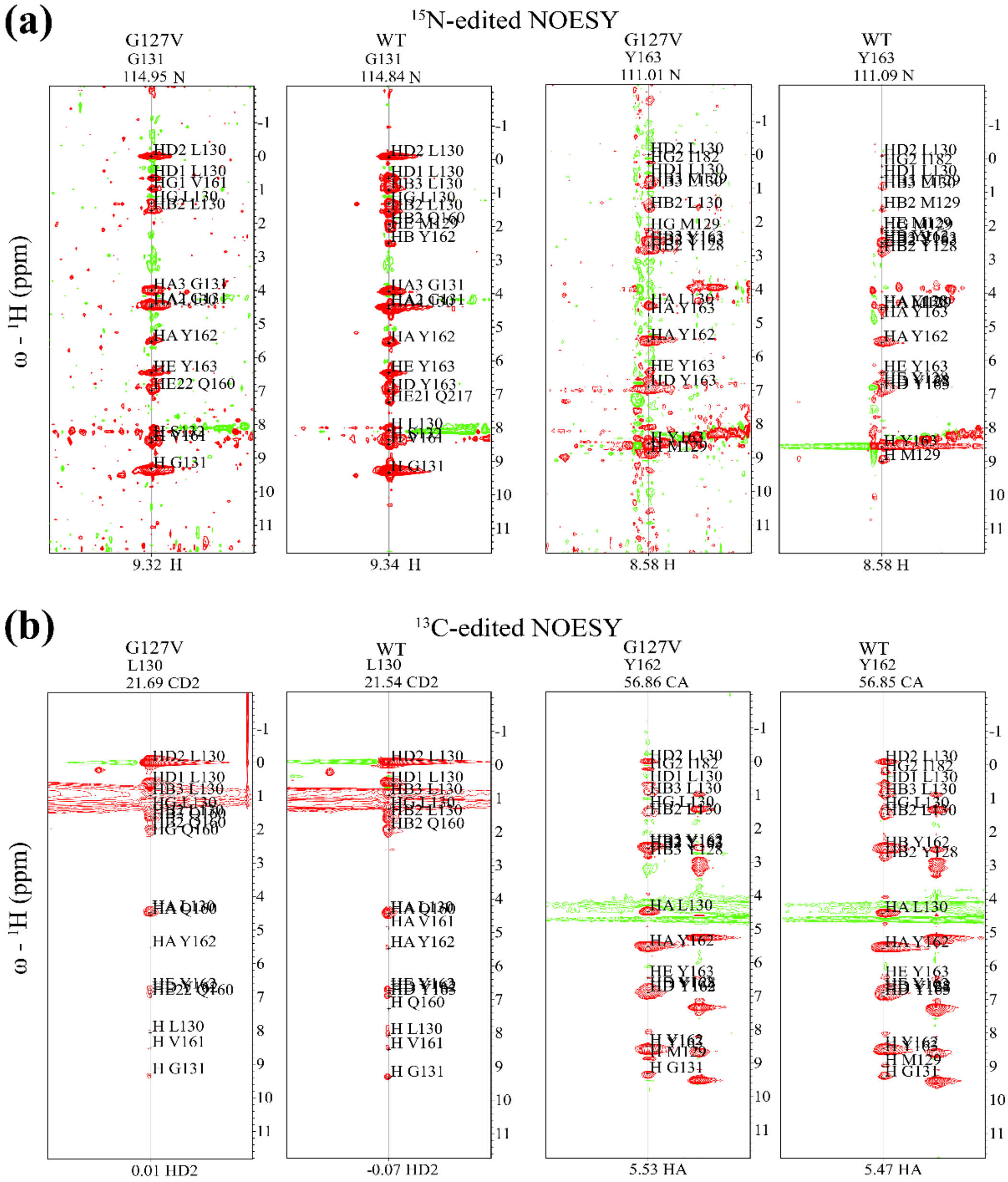

Figure S4. A comparison of the primary differential peak intensities of 3D ^{15}N-edited NOESY-HSQC and ^{13}C-edited NOESY-HSQC spectra between HuPrP(G127V) and WT HuPrP. Several NOESY peaks, such as HN/G131-HA/Y162, HN/Y163-HD2/L130, HD2/L130-HE/Y163, and HA/Y162-HD2/L130, of the mutant are somewhat weaker than those of the WT protein. More importantly, some NOESY peaks of the mutant, such as HN/G131-HB/Y162, HD2/L130-HN/V161, and HD2/L130-HA/Y162, are missing. Additionally, the HN/Y128-related NOESY peaks are invisible in the mutant because of a lack of HN/Y128 assignment, whereas these peaks are visible in the WT. These NMR spectra were analysed using CARA software.

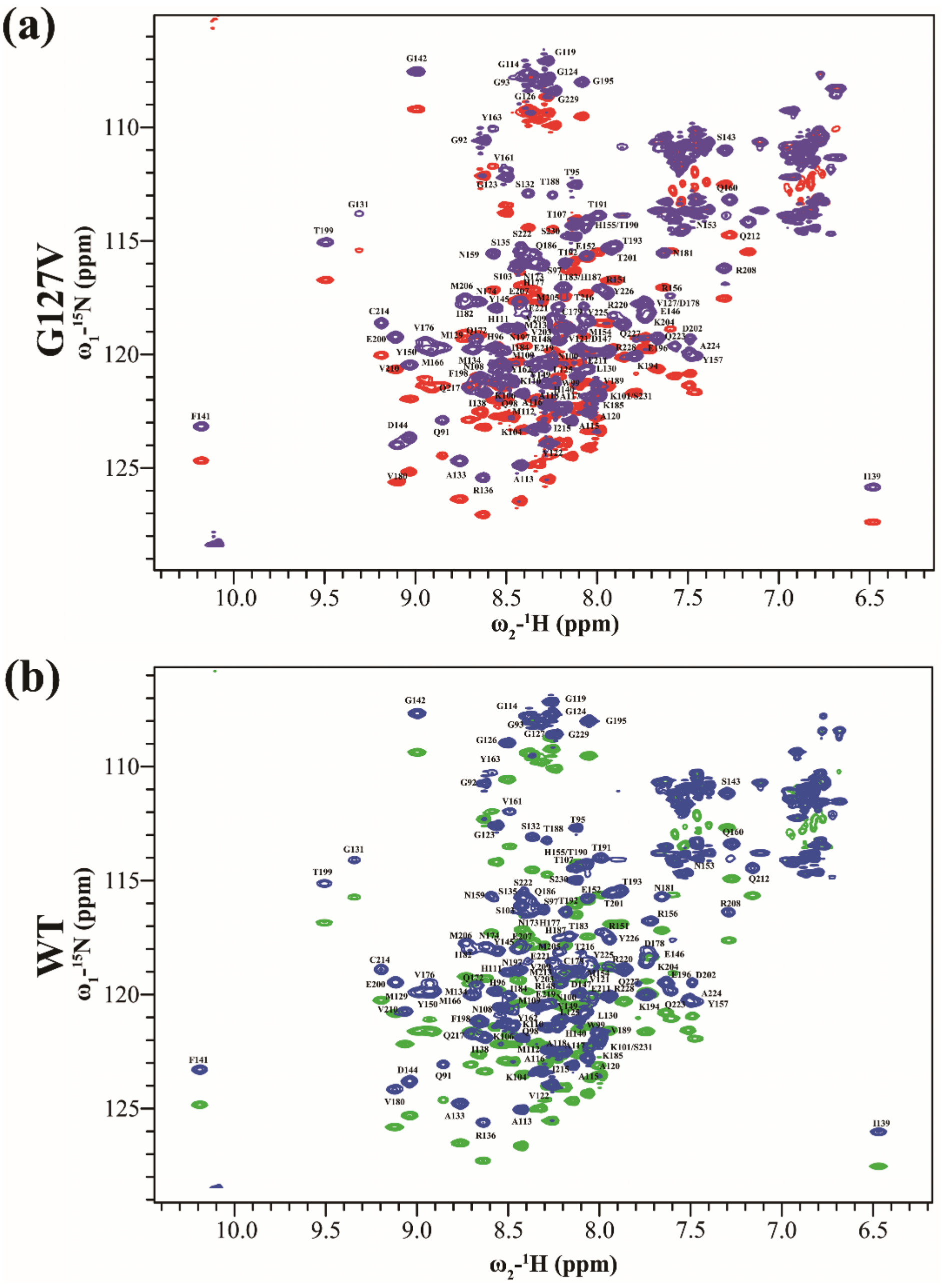

Figure S5. 2D ^{1}H-^{15}N IPAP-HSQC spectra recorded for HuPrP(G127V) and WT HuPrP in order to measure RDCs of the backbone amide groups. (a) HuPrP(G127V); (b) WT HuPrP. The in-phase spectra are purple for the mutant and blue for the WT protein and the anti-phase spectra are red for the mutant and green for the WT protein.

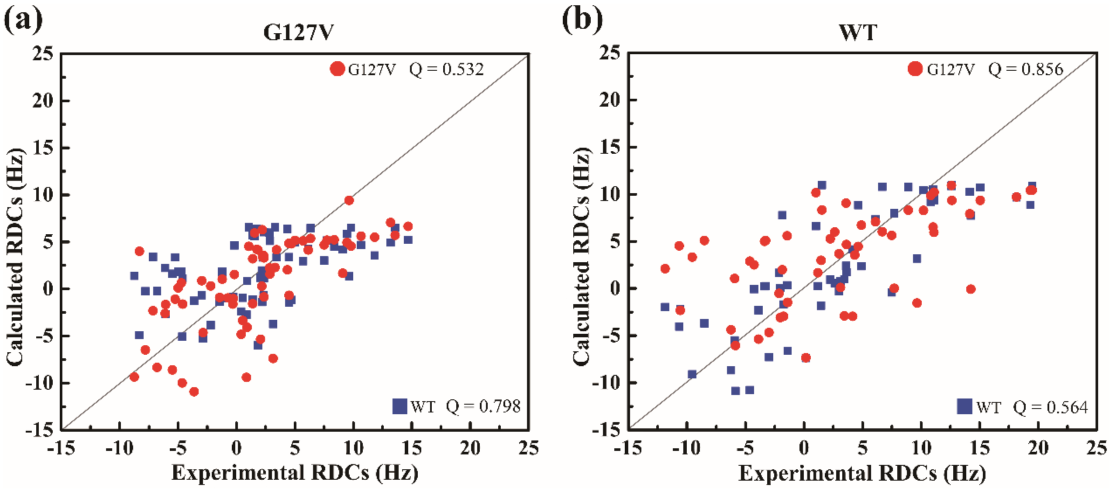

Figure S6. Correlation plots of experimental RDCs measured vs. theoretical RDCs for HuPrP(G127V) and WT HuPrP. The experimental RDCs were measured from 2D ^{1}H-^{15}N IPAP-HSQC spectra recorded for either the mutant (a) or WT protein (b). The theoretical RDCs were calculated from 3D structures of both the mutant (PDB 5YJ4, red) and WT protein (PDB 5YJ5, blue). As indicated by the calculated Q-values, the experimental RDCs for HuPrP(G127V) were fitted better to the structure of the mutant (Q = 0.532) than to that of the WT protein (Q = 0.798). Similarly, the experimental RDCs of the WT protein were fitted better to the structure of the WT protein (Q = 0.564) than to that of the mutant protein (Q = 0.856). Notably, we only used the experimental RDCs of the well-resolved residues located in the C-terminal structural cores of the two proteins.

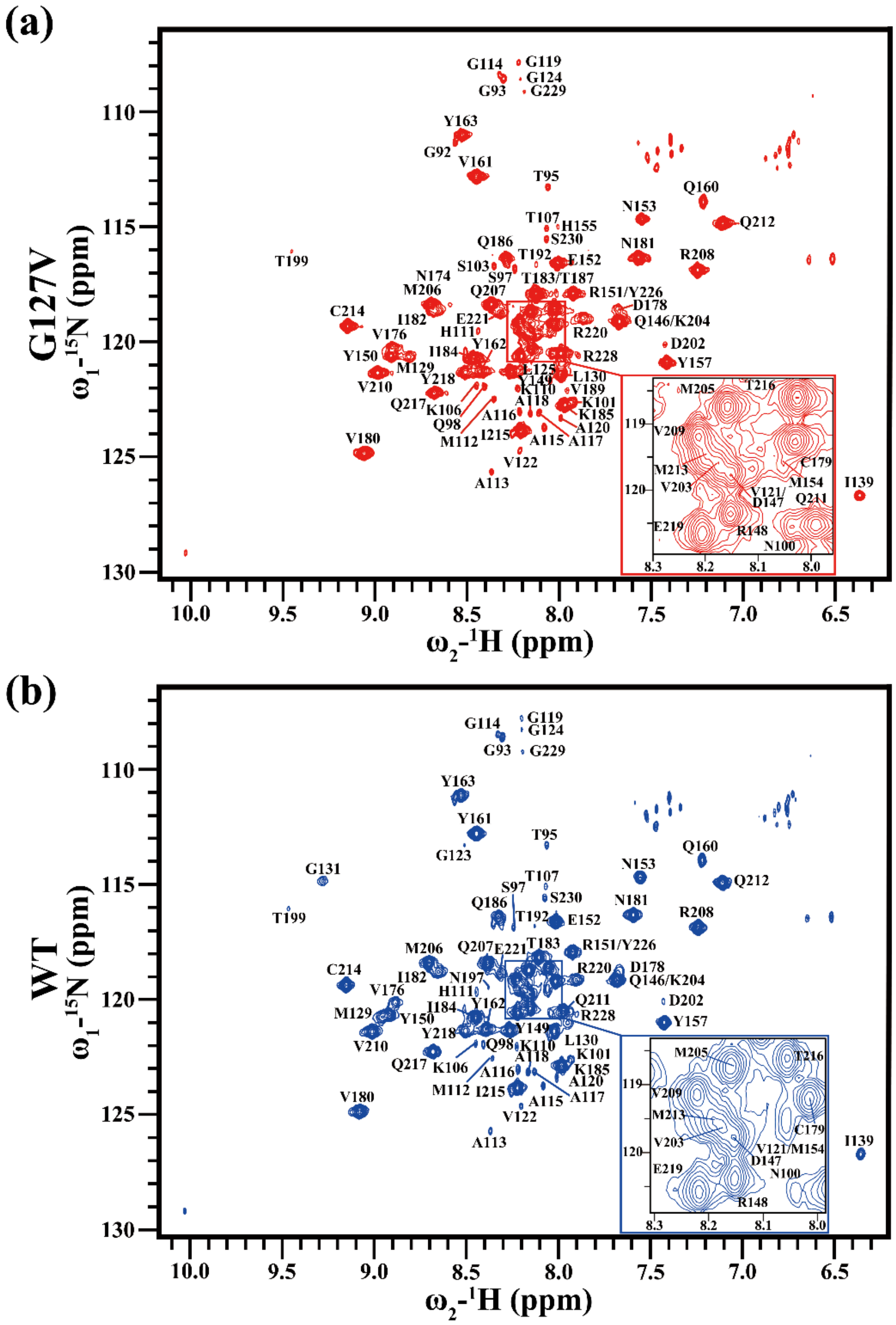

Figure S7. 2D Fast ^{1}H-^{15}N HSQC spectra for HuPrP(G127V) (red) and WT HuPrP in order to observe peak weakening caused by H/D exchange. Both proteins were re-dissolved in D_2O buffer for three hours.

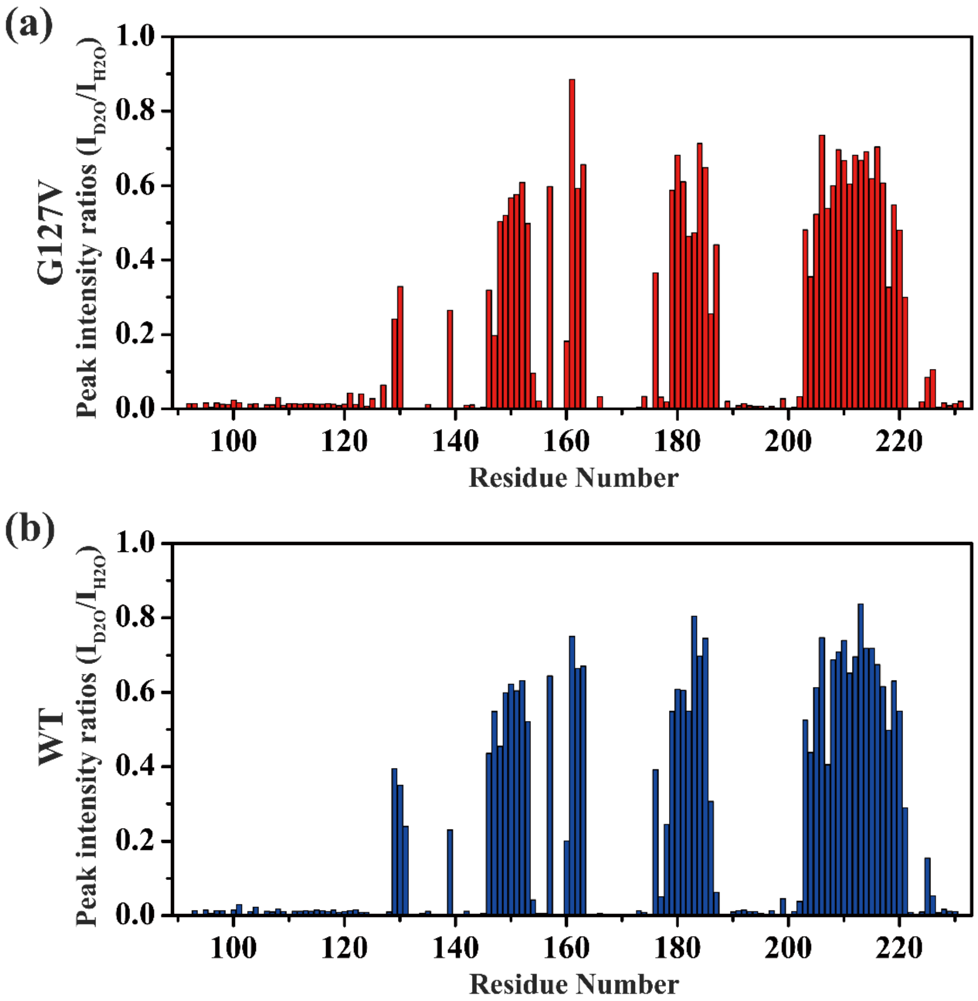

Figure S8. Peak intensity ratios of Fast ^{1}H-^{15}N HSQC spectra recorded for HuPrP(G127V) (red) and WT HuPrP (blue) re-dissolved in D_2O buffer for three hours and those dissolved in H_2O buffer. The amide proton of Gly131 in the mutant underwent a complete H/D exchange and became invisible in the HSQC spectrum. The amide proton of Met154 and His 155 in the mutant became more stable than WT protein.

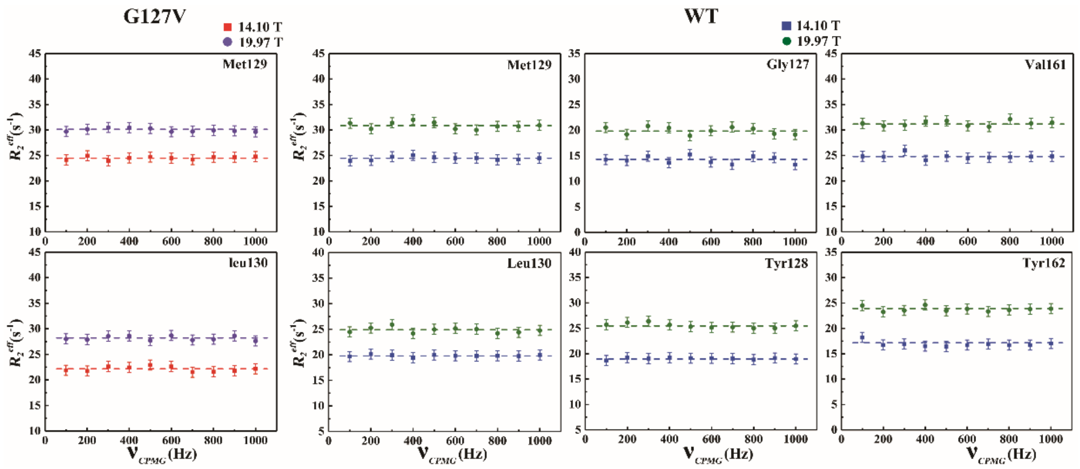

Figure S9. ^{15}N CPMG RD analysis for the SSs in HuPrP(G127V) and the β-sheet in WT HuPrP. Met129 and Leu130, located at the SSs in the mutant, did not exhibit significant conformational exchanges. No residues located within the β-sheet in the WT protein showed observable conformational exchanges. All CPMG RD experimental data were acquired at magnetic field strengths of 14.10 T (red for G127V, violet for WT) and 19.97 T (blue for G127V, olive for WT).

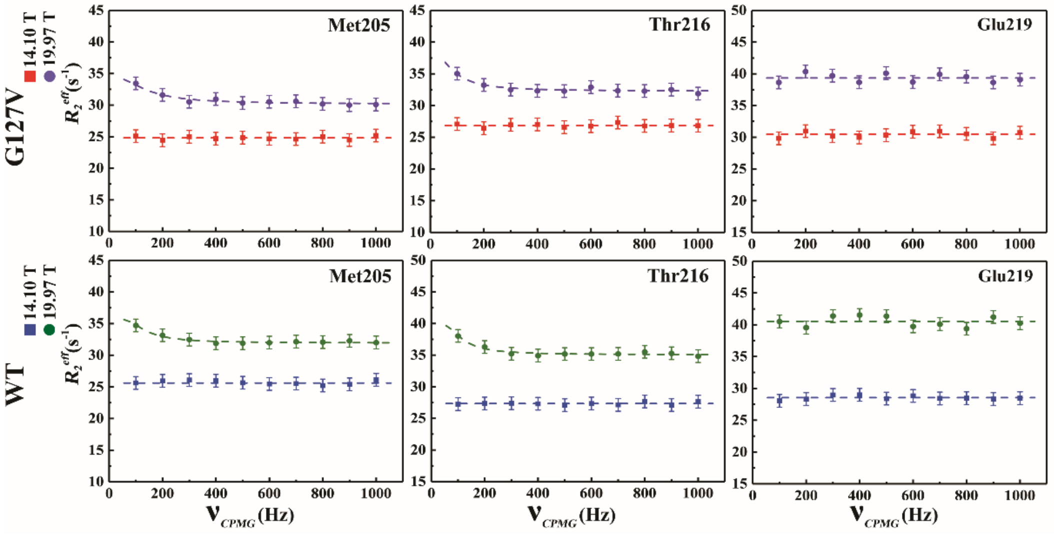

Figure S10. ^{15}N CPMG RD analysis for residues located within the α3 helix in either HuPrP(G127V) or WT HuPrP. All CPMG RD experimental data were acquired at magnetic field strengths of 14.10 T (red for G127V, violet for WT) and 19.97 T (blue for the mutant, olive for WT). Met205 and Thr216 in both proteins exhibited more significant conformational exchanges at 19.97 T than those at 14.10 T. Similarly, both residues showed larger R_2/R_1 ratios at 19.97 T than those at 14.10 T (Figure 3). Glu219 in both proteins displayed unobservable conformational exchanges at the two magnetic field strengths, although they showed large R_2 and J(0) values.

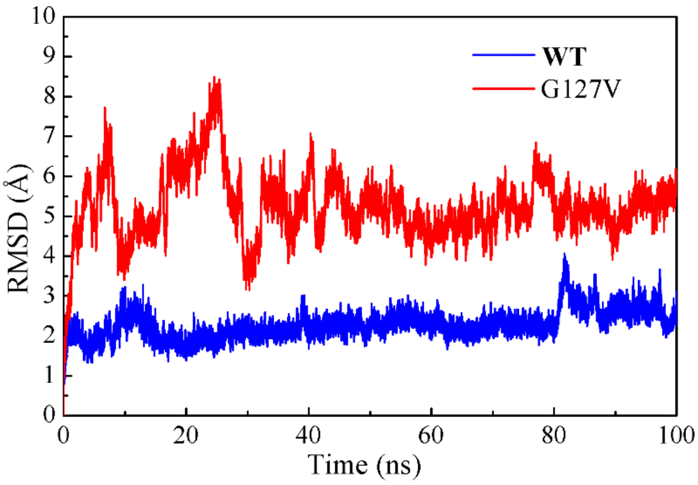

Figure S11. Time evolutions of the RMSDs for the determined 3D structures of HuPrP(G127V) and WT HuPrP obtained from MD simulations. The RMSDs of the mutant were much larger than those of the WT protein, which suggests that the mutant underwent a more significant structural fluctuation than the WT protein and may partially account for the somewhat larger backbone atomic RMSD of the mutant compared with that of the WT protein (Table S1, 1.28 ± 0.27 Å vs. 0.99 ± 0.21 Å).

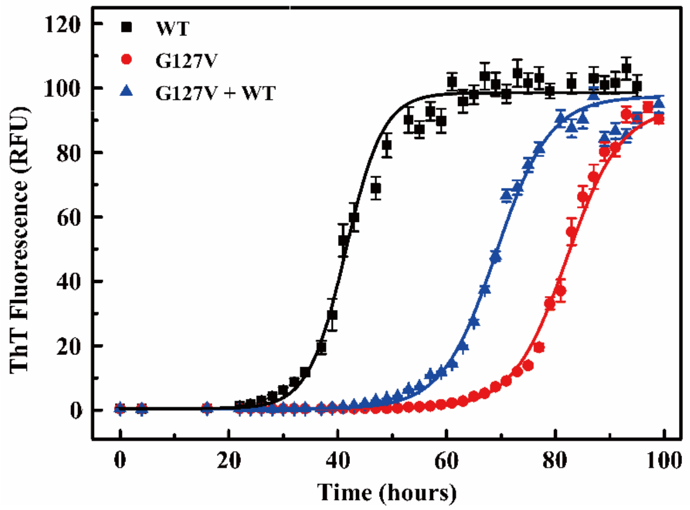

Figure S12. The G127V mutant slows down the fibrillization rate of HuPrP. The lag phases (mean ± SD) of WT HuPrP (100 μM) and HuPrP(G127V) (100 μM) were 25 ± 2 h (black square) and 61 ± 2 h (red dot) measured from three repeated experiments, respectively. The mixing sample of WT HuPrP and HuPrP(G127V) (50 μM : 50 μM) exhibited a fibrillization rate slower than WT HuPrP but faster than HuPrP(G127V), and the lag phase was 47 ± 2 h (blue triangle). All fibrillization experiments were implemented in Fibrillization buffer (2 M GdnHCl, 50 mM Tris, pH 7.4) with 220 rpm at 37 °C. All fibrillization rates were monitored by thioflavine T fluorescence with emission at 482 nm upon excitation at 440 nm on Multimode Plate Reader EnSpire (PerkinElmer, Inc.) at pH 7.4 and 37 °C. The cures were fitted with Boltzmann function on Origin.
